# Deep learning reveals a neurocomputational mechanism predicting depression risk in adolescents

**DOI:** 10.1126/sciadv.aed0772

**Published:** 2026-08-07

**Authors:** Han Lu, Xiaoqian Yan, Benjamin Becker, Andreas Heinz, Barbara J. Sahakian, Christelle Langley, Zhaoyu Zuo, Luolong Cao, Zuo Zhang, Lauren Robinson, Nilakshi Vaidya, Jeanne Winterer, Sinead King, Charlotte Walton, Tobias Banaschewski, Gareth J. Barker, Arun L.W. Bokde, Rüdiger Brühl, Herta Flor, Hugh Garavan, Penny Gowland, Antoine Grigis, Herve Lemaitre, Jean-Luc Martinot, Marie-Laure Paillère Martinot, Eric Artiges, Frauke Nees, Dimitri Papadopoulos Orfanos, Luise Poustka, Hedi Kebir, Ulrike Schmidt, Julia Sinclair, Michael N. Smolka, Sarah Hohmann, Nathalie Holz, Henrik Walter, Robert Whelan, Sylvane Desrivières, Gunter Schumann, Qiang Luo, STRATIFY/ESTRA Consortium, IMAGEN Consortium

**Affiliations:** ^1^ School of Artificial Intelligence, Shenzhen University, Shenzhen, China.; ^2^ Institute of Science and Technology for Brain-Inspired Intelligence, Fudan University, Shanghai, China.; ^3^ MIND & AI Lab, Department of Psychology, The University of Hong Kong, Hong Kong SAR, China.; ^4^ SRT AI, Society & Social Dynamics, Faculty of Social Sciences, The University of Hong Kong, Hong Kong SAR, China.; ^5^ Department of Psychiatry and Psychotherapy, University of Tübingen and German Center for Mental Health (DZPG), Site Tübingen, Germany.; ^6^ Department of Psychiatry and Psychotherapy, Technische Universität Dresden, Dresden, Germany.; ^7^ Department of Psychiatry, University of Cambridge, Cambridge CB2 0SZ, UK.; ^8^ Behavioural and Clinical Neuroscience Institute, Department of Psychology, University of Cambridge, Cambridge CB2 3EB, UK.; ^9^ Social Genetic and Developmental Psychiatry Centre, Institute of Psychiatry, Psychology, and Neuroscience, King’s College London, London SE5 8AF, UK.; ^10^ School of Psychology, Institute for Mental Health, University of Birmingham, Birmingham, UK.; ^11^ Oxford Institute of Clinical Psychology Training and Research, Oxford University, Oxford, UK.; ^12^ Centre for Population Neuroscience and Stratified Medicine (PONS), Department of Psychiatry and Psychotherapy, Charité Universitätsmedizin Berlin, Germany.; ^13^ Charité—Universitätsmedizin Berlin, corporate member of Freie Universität Berlin, Humboldt-Universität zu Berlin, and Berlin Institute of Health, Department of Psychiatry and Psychotherapy, Campus Charité Mitte, Charitéplatz 1, Berlin, Germany.; ^14^ Department of Education and Psychology, Freie Universität Berlin, Berlin, Germany.; ^15^ Psychology Department, B44 University Road, University of Southampton, Southampton SO17 1PS, UK.; ^16^ Department of Child and Adolescent Psychiatry and Psychotherapy, Central Institute of Mental Health, Medical Faculty Mannheim, Heidelberg University, Square J5, 68159 Mannheim, Germany; German Center for Mental Health (DZPG), partner site Mannheim-Heidelberg-Ulm.; ^17^ Department of Neuroimaging, Institute of Psychiatry, Psychology, and Neuroscience, King’s College London, UK.; ^18^ Discipline of Psychiatry, School of Medicine and Trinity College Institute of Neuroscience, Trinity College Dublin, Dublin, Ireland.; ^19^ Physikalisch-Technische Bundesanstalt (PTB), Braunschweig and Berlin, Germany.; ^20^ Institute of Cognitive and Clinical Neuroscience, Central Institute of Mental Health, Medical Faculty Mannheim, Heidelberg University, Square J5, Mannheim, Germany.; ^21^ Department of Psychology, School of Social Sciences, University of Mannheim, 68131 Mannheim, Germany.; ^22^ Departments of Psychiatry and Psychology, University of Vermont, Burlington, VT 05405, USA.; ^23^ Sir Peter Mansfield Imaging Centre School of Physics and Astronomy, University of Nottingham, University Park, Nottingham, UK.; ^24^ NeuroSpin, CEA, Université Paris-Saclay, F-91191 Gif-sur-Yvette, France.; ^25^ Institut des Maladies Neurodégénératives, UMR 5293, CNRS, CEA, Université de Bordeaux, 33076 Bordeaux, France.; ^26^ Institut National de la Santé et de la Recherche Médicale, INSERM U1299 “Trajectoires développementales en psychiatrie”; Université Paris-Saclay, Ecole Normale supérieure Paris-Saclay, CNRS, Centre Borelli, Gif-sur-Yvette, France.; ^27^ Research Department, LABD-PSY, Etablissement Public de Santé (EPS) Barthélemy Durand, 91700 Sainte-Geneviève-des-Bois, France.; ^28^ AP-HP, Sorbonne Université, Department of Child and Adolescent Psychiatry, Pitié-Salpêtrière Hospital, Paris, France.; ^29^ Institute of Medical Psychology, Ludwig-Maximilians-Universität (LMU) in Munich, Munich, Germany.; ^30^ Department of Child and Adolescent Psychiatry, Center for Psychosocial Medicine, University Hospital Heidelberg, Heidelberg, Germany.; ^31^ German Center for Mental Health (DZPG), Site Berlin-Potsdam, Germany.; ^32^ Department of Psychological Medicine, Section for Eating Disorders, Institute of Psychiatry, Psychology and Neuroscience, King’s College London, London SE5 8AF, UK.; ^33^ South London and Maudsley NHS Foundation Trust, London, UK.; ^34^ Clinical and Experimental Sciences, Faculty of Medicine, University of Southampton, Southampton, UK.; ^35^ Department of Psychiatry and Psychotherapy CCM, Charité—Universitätsmedizin Berlin, corporate member of Freie Universität Berlin, Humboldt-Universität zu Berlin, and Berlin Institute of Health, Berlin, Germany.; ^36^ School of Psychology and Global Brain Health Institute, Trinity College Dublin, Ireland.; ^37^ Research Institute of Intelligent Complex Systems, Fudan University, Shanghai, China.; ^38^ State Key Laboratory of Medical Neurobiology and MOE Frontiers Center for Brain Science, Institutes of Brain Science, Fudan University, Shanghai, China.

## Abstract

Early detection and prevention of psychiatric disorders, particularly depression, remain as major global health challenges, yet reliable tools for identifying individuals before symptom onset are lacking. Here, we combine functional neuroimaging with computational modeling to identify a mechanistic biomarker of depression risk. In a population-based adolescent cohort (IMAGEN, *N* = 1332), we found that weakened neural representations of emotional signals were linked to depressive symptoms. Perturbation experiments in a brain-aligned deep learning model showed that this deficit reflects overregularized emotion perception, producing a negative perceptual bias. A neurocomputational signature of this mechanism predicted depression symptom onset up to 4 years later at the IMAGEN follow-up (*N* = 725), was associated with both a genetic-risk variant and polygenic risk for depression, and improved depression classification in a patient cohort (STRATIFY, *N* = 411). These findings suggest a possible mechanism linking genetic vulnerability to altered emotion perception and future depression, and propose a predictive computational marker with potential for early detection and prevention.

## Teaser

A neurocomputational signature predicts vulnerability to depression from altered emotion processing.

## INTRODUCTION

Major depressive disorder (MDD), which afflicts more than 332 million of the world population (*1*), is often difficult to treat once fully established (*2*) and is highly prone to relapse (*3*). Consequently, the prevention and early detection have emerged as global health priorities (*4*). While association-based studies of the human brain have cataloged numerous neural correlates (*5*), the field suffers from a crucial bottleneck: the absence of mechanistically interpretable biomarkers that can predict individual risk (*6*). Bridging the gap between descriptive associations and computational mechanisms is essential to transform psychiatric nosology and enable preemptive prevention.

Deficient emotional processing is a hallmark feature of depression (*7*) and a promising candidate marker for illness vulnerability (*8*). However, progress has been limited due to the lack of mechanistic understanding of these deficits. Leading theoretical frameworks, such as the theory of constructed emotion (*9*), posit that conceptual knowledge from memory systems shapes sensory coding in the visual hierarchy (*9*). Neuroimaging studies support this view, showing that emotional categories can be decoded from visual cortex activity (*10*–*12*), and its dysfunction during negative facial emotion processing is linked to depression (*13*, *14*). We focused specifically on angry facial expressions as they serve as potent signals of social threat and rejection. Altered processing of such social cues is central to the interpersonal difficulties and negative processing biases often observed in depression (*15*). More recently, the brain’s responses to angry faces have been decomposed into two networks, an orbitofrontal-related network and an occipital-related network, both of which are linked to the resilience to developing emotional disorders following childhood adversity (*8*). Nevertheless, these observations remain fundamentally correlative, leaving a knowledge gap: How are emotion-specific visual representations computationally formed through learning, and how does the disruption of this process confer vulnerability to disease?

To resolve this gap, we introduce a perturbation-based neurocomputational framework. We instantiate a dual-pathway deep neural network (DNN) (*16*) in which a “conceptual pathway” encodes abstract emotional concepts that then regulate learning in a “perceptual network.” Conceptually, “regularization” here refers to the process where the brain uses abstract knowledge to guide and constrain how it processes raw visual information. Ideally, this helps us recognize facial emotions quickly. However, if this guidance becomes too rigid (i.e., “overregularized”), perception may become biased by negative concepts rather than accurately reflecting the visual reality. This architecture provides a computational testbed: By systematically perturbing the strength of the conceptual regularization in the model, we can quantitatively simulate the emergence of both normative and pathological emotion representations. We then directly test this model against human neuroimaging data, evaluating the alignment between DNN-derived representations and brain activity during the perception of angry faces (*17*). This approach moves beyond traditional correlational neuroscience to propose a theoretical link between a defined computational mechanism and depression risk, thereby suggesting a previously unrecognized class of candidate mechanistic markers for preemptive psychiatry.

In this study, we investigated neurocomputational mechanisms underlying visual emotion processing and its pathological changes in depression by addressing the following questions: (i) Are pathological changes in the brain’s visual processing of anger associated with emotional symptoms? (ii) Can nonoptimal regularization, as a computational mechanism, reproduce these pathological changes? (iii) Derived from this mechanism, can a computational marker predict future symptom onset? (iv) Is the computational marker associated with depression-related genetic risk and persistent in patients with depression?

## RESULTS

### Study design

**Fig. 1.**
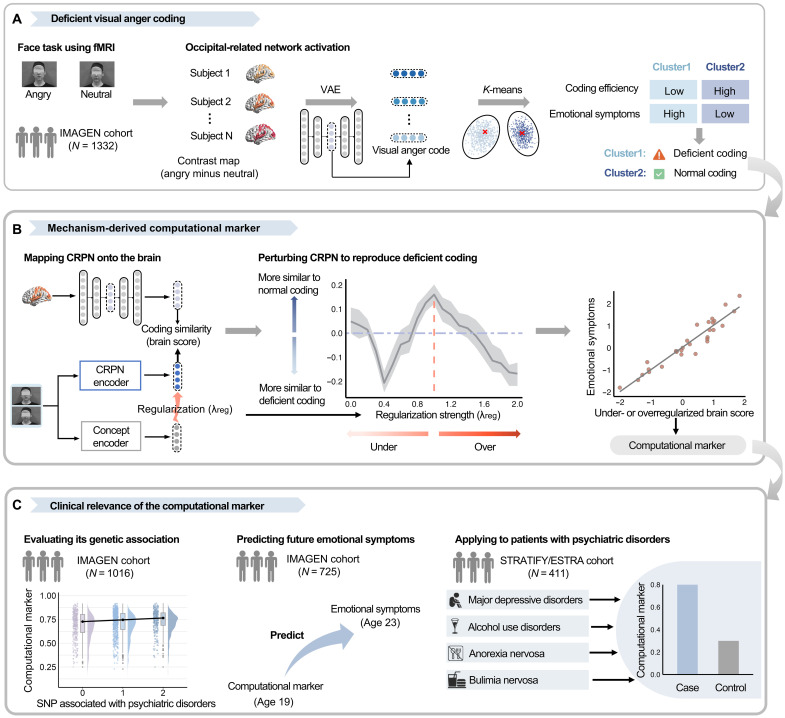
Data analysis flowchart. (**A**) We extracted the visual anger code for each participant at age 19 years in the IMAGEN cohort by applying a VAE to the occipital-related network activation in response to angry faces. Subsequently, we clustered these visual anger codes and compared different clusters to explore their link to coding efficiency and emotional symptoms (see Materials and Methods). (**B**) After mapping the CRPN code onto the brain’s visual anger code, we perturbed the concept-regularization strength to test whether under- or overregularization could serve as a computational marker of deficient visual anger coding. (**C**) We assessed the predictive value, genetic relevance, and applicable value of the computational marker. The subject’s eyes are masked to protect privacy.

We first analyzed the functional magnetic resonance imaging (fMRI) data collected during an emotional face task at age 19 years in the IMAGEN cohort (*N* = 1332, 52.85% girls; table S1) to extract the visual anger code for each participant, testing the association between deficient visual coding and emotional symptoms. Second, we tested whether the dual-pathway DNN model (*16*) can reproduce neural representations of angry faces by assessing the similarity between the DNN-derived code and the brain’s visual code. Next, we systematically perturbed the concept-regularization strength in our dual-pathway framework to identify the pathological strength level that can regenerate the deficient visual coding. Third, we derived a computational marker from the concept-regularization mechanism and demonstrated its clinical relevance, that is, specific to depression, including (i) its predictability of the onset of emotional symptoms at age 23 years; (ii) its associations with depression-related genetic risk; and (iii) its persistence in an independent clinical cohort (Fig. 1).

### Weakened visual anger code associated with emotional symptoms

**Fig. 2.**
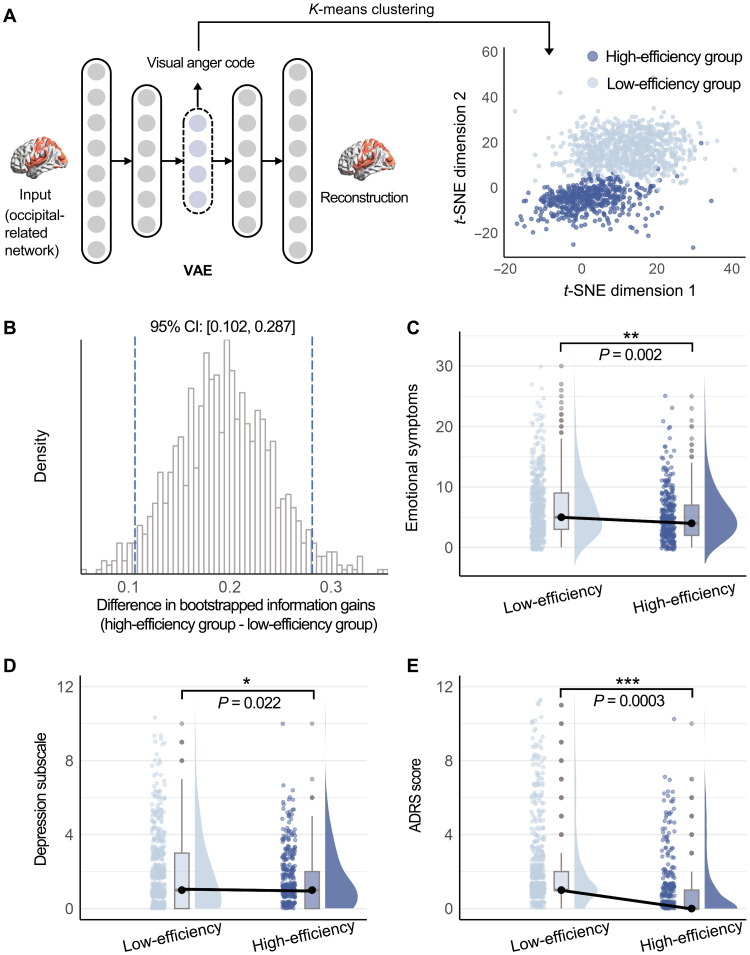
Two distinct patterns of the brain’s visual anger code. (**A**) VAE was used to learn the lower-dimensional latent representation for clustering of the individuals into two distinct patterns of visual anger code, which were visualized using t-distributed stochastic neighbor embedding (t-SNE). (**B**) The high-efficiency group (*N* = 485) exhibited more efficient information encoding than the low-efficiency group (*N* = 847). (**C** to **E**) Individuals in the high-efficiency group had lower levels of emotional symptoms, depressive scores, and ADRS scores compared to those in the low-efficiency group.

To extract the visual codes of angry faces (i.e., the low-dimensional latent representations) from the highly redundant voxel-wise brain activation data (*18*, *19*), we applied a variational autoencoder (VAE) to the occipital-related network activation in response to angry faces (i.e., the anger minus neutral contrast map) (*8*). This resulted in a 256-dimensional visual anger code for each participant at age 19 years. Clustering analysis using *k*-means revealed two distinct clusters of these visual anger codes (Fig. 2A): a high-efficiency group with normal coding (*N* = 485) and a low-efficiency group with deficient coding (*N* = 847). Compared with the high-efficiency group, the low-efficiency group exhibited reduced coding efficiency, as quantified by lower information gain (∆IG = −0.211; 95% confidence interval, CI = [−0.287, −0.102]) (Fig. 2B), and showed more emotional symptoms (Cohen’s *d* = 0.23, β = 0.178, 95% CI = [0.064, 0.293], *P* = 0.002; Fig. 2C), as measured by the composite score of depression, generalized anxiety disorder, specific phobia, and eating disorder subscales (see Materials and Methods for details). In line with this inverse relationship observed between groups, we observed a significant negative association between individual coding efficiency and emotional symptoms (β = −0.063, 95% CI = [−0.121, −0.004], *P* = 0.035).

### Sensitivity analysis

Using the development and well-being assessment (DAWBA) (*20*), we showed that the low-efficiency group was more likely to receive a diagnosis of an emotional disorder as compared with the high-efficiency group (7.23 versus 2.27%; $\chi_{1}^{2}=11.226$, *P* = 0.0008). In particular, this difference was significant in the depression subscale (Cohen’s *d* = 0.19, β=0.146,95%CI=[0.020,0.272],P=0.022; Fig. 2D) and was confirmed by another rating scale (i.e., the adolescent depression rating scale, ADRS; Cohen’s *d* = 0.23, β=0.219,95%CI=[0.101,0.336],P=0.0003; Fig. 2E). These two groups did not differ in terms of the sex, handedness, data collection site, socioeconomic status, or head motion [mean framewise displacement (FD)]. The above findings were specific to angry face processing, as we detected no significant association between emotional symptoms and the visual happy code, which were extracted in a similar way by the VAE (see Supplementary Text).

To evaluate the generalizability of the identified subgroups and rule out potential overfitting, we randomly divided participants into training (70%) and test (30%) sets with stratification by data collection site. The VAE and clustering solution were derived exclusively from the training set, while cluster stability and group differences were evaluated in the independent test set (see Supplementary Text). Under this procedure, the pattern observed in the full sample was replicated in the held-out test set, with the low-efficiency group showing significantly reduced coding efficiency (∆IG = −0.126, 95% CI = [−0.229, −0.015]) and elevated emotional symptoms (β = 0.221, 95% CI = [0.012, 0.429], *P* = 0.038) compared with the high-efficiency group, indicating the robustness of the findings obtained from the full sample.

### Conceptual regularization reproduced visual anger code

**Fig. 3.**
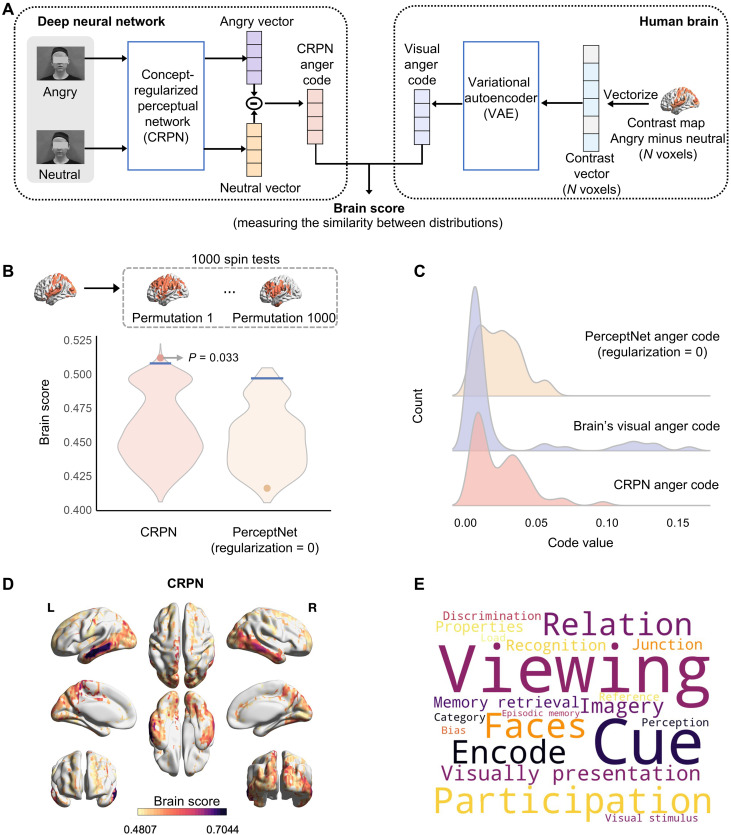
Quantifying similarity of dual-pathway DNN to human. (**A**) Mapping between CRPN code and human brain’s visual anger code according to their distributions. (**B**) The CRPN code showed a significant mapping onto the brain’s visual anger code via 1000 spin tests. Points represent the brain scores for both CRPN and PerceptNet codes. Blue lines represent *P* = 0.05. (**C**) Density plot. The brain’s visual anger code, CRPN code, and PerceptNet anger code were normalized and converted to distributions. Jensen-Shannon divergence was used to measure their similarity. The CRPN code significantly improved this brain mapping compared to the PerceptNet anger code without concept regularization. (**D**) Voxel-wise exploratory analysis for each voxel (i.e., a six-voxel-radius sphere centered at this voxel). The CRPN had high similarities in some frontal voxels, where abstract concepts were mainly represented. Only voxels with brain scores higher than the average precentral brain score were displayed. L, left; R, right. (**E**) NeuroSynth decoding of (D). The word cloud displays the top 20 functional terms based on their correlation coefficients for (D).

We found a significant similarity, quantified by the brain score (see Materials and Methods), between the brain’s visual anger code derived from the occipital-related network and the dual-pathway DNN’s anger code (*S* = 0.512, *P* = 0.033 by 1000 spin tests; Fig. 3, A and B). To validate the specificity of this mapping, we compared it against the orbitofrontal-related network (*8*), which is also engaged in angry face processing but supports higher-level conceptual functions. This analysis revealed that the DNN’s correspondence to the occipital-related network was significantly stronger than its correspondence to the orbitofrontal-related network (∆*S* = 0.206, 95% CI = [0.158, 0.265]). Together, these results indicate that the dual-pathway DNN predominantly captures visual-perceptual components of angry face processing, consistent with previous work establishing DNNs as models of visual system (*21*, *22*).

Building on this similarity, we found that the dual-pathway DNN code explained 0.68 of the maximum explainable signals in the brain’s visual anger code estimated by the noise ceiling analysis. This similarity was also significantly greater than that with the traditional DNN (*16*) without the concept regularization (∆*S* = 0.096, 95% CI = [0.028, 0.118]; Fig. 3C), and this advantage was robust across all data collection sites (table S2).

Exploratorily, we conducted a voxel-level mapping of the dual-pathway DNN code. We found high similarities at many occipital voxels and some frontal voxels (Fig. 3D). The distribution of these voxels can be associated with both perceptual (e.g., viewing and faces) and conceptual (e.g., imagery and memory retrieval) processes by the NeuroSynth-based meta-analytic decoding (Fig. 3E) (*23*).

### Overregularization regenerated deficient visual code and negative emotion bias

**Fig. 4.**
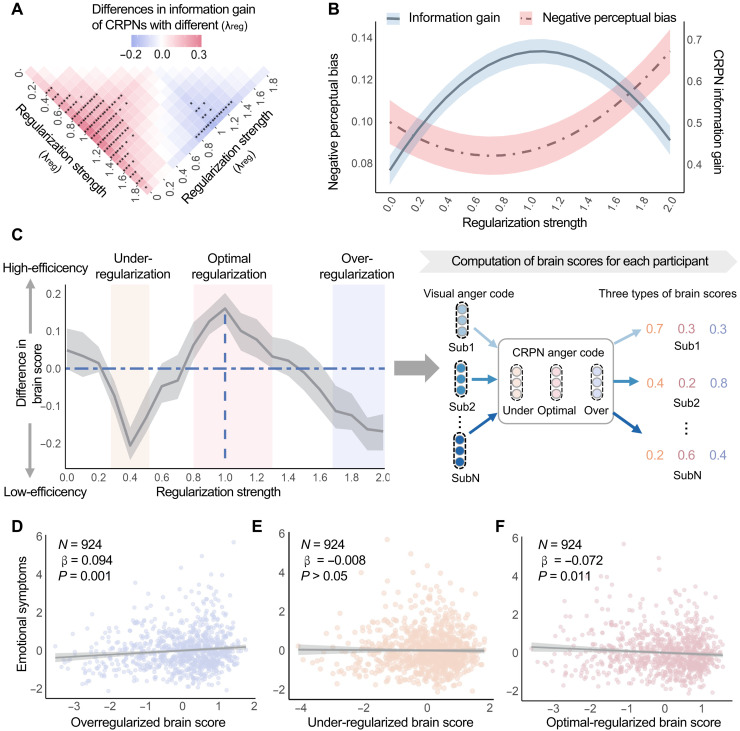
Overregularization mechanism and its computational marker. (**A**) ANOVA reveals significant differences in the information gain of CRPN code under different $\lambda_{\text{reg}}$:. *P < 0.05; **P < 0.01; ***P < 0.001. (**B**) The plot shows the information gain and negative perceptual bias as a function of $\lambda_{\text{reg}}$. Due to the use of different random seeds for CRPN training, five measurements were taken at each value of $\lambda_{\text{reg}}$. For both metrics, a linear mixed-effects model with a quadratic term based on $\lambda_{\text{reg}}$ was fitted. The plot displays the fitted values along with their 95% CIs. (**C**) Based on the $\lambda_{\text{reg}}$ values associated with significant differences in brain scores between the two groups, we defined under-, optimal, and overregularization. Shaded gray area indicates 95% CI. For each condition, we computed the brain score between the CRPN code and visual anger code for each participant at age 19 years. (**D** to **F**) Both the optimal and the overregularized brain scores were associated with emotional symptoms, but their associations went to different directions. Residual *z*-scores were used after controlling for the covariates (Materials and Methods).

In the perturbation experiment of the concept-regularization strength in the dual-pathway DNN ($\lambda_{\text{reg}}$ from 0 to 2; see Materials and Methods), we found that the information gain of the DNN code varied significantly with the strength level ($\eta_{p}^{2}$ = 0.776, *F* = 14.57, *P* < 0.001; Fig. 4A) and peaked at an intermediate regularization level ($\lambda_{\text{reg}}$ = 1; Fig. 4B), where the DNN had achieved its best performance of emotion recognition (*16*). Meanwhile, we found that negative emotion bias [i.e., the misclassification of neutral faces as angry or sad; (*24*)] increased as stimulus strength deviated from the intermediate level toward the extremes, with the strongest increase observed at higher strength levels (Fig. 4B). Comparing with the brain’s visual anger code extracted above, the DNN codes within an optimal-regularization range [$\lambda_{\text{reg}}\in \left(0.8,1.3\right)$] were more similar to the high-efficiency group’s code (with less emotional symptoms) and less similar outside this range, relative to the low-efficiency group (with more emotional symptoms) (Fig. 4C and table S3).

### Computational marker associated with emotional symptoms at age 19 years

Among the IMAGEN individuals at age 19 years, we found that higher brain scores related to the DNN code with overregularization, named overregularized brain scores, were associated with more emotional symptoms (β = 0.094, 95% CI = [0.038, 0.151], *P* = 0.001; Fig. 4D), after adjusting for data collection site, sex, handedness, and socioeconomic status. In contrast, brain scores related to under-regularization showed no such association (Fig. 4E). Consistently, we also found that higher brain scores related to the DNN code with optimal regularization, called optimal-regularized brain scores, were associated with fewer emotional symptoms (β = −0.072, 95% CI = [−0.128, −0.016], *P* = 0.011; Fig. 4F). Using another rating scale (i.e., the ADRS total score), we confirmed these associations (overregularization: β = 0.079, 95% CI = [0.021, 0.136], *P* = 0.008; optimal regularization: β = −0.073, 95% CI = [−0.131, −0.016], *P* = 0.012).

Crucially, these findings were robust to potential confounds related to in-scanner behavior. First, the results could not be merely explained by visual inattention, as we found no significant correlations between sustained attention performance [as assessed by a rapid visual information processing task; (*25*)] and the brain scores. Second, we ruled out the influence of head motion. Mean FD was not significantly associated with the derived brain scores. Furthermore, when mean FD was included as an additional covariate, both the optimal- (β = −0.081, 95% CI = [−0.138, −0.024], *P* = 0.005) and overregularized (β = 0.084, 95% CI = [0.026, 0.141], *P* = 0.005) brain scores continued to show significant associations with emotional symptoms. In addition, while brain scores differed significantly by data collection site, these differences did not drive the main results: We found no significant interaction effects between brain scores and data collection site on emotional symptoms.

To further examine the sex-specific effects, we conducted sex-stratified sensitivity analyses. Separate VAEs were trained for males (*N* = 628) and females (*N* = 704) using their respective unadjusted contrast maps, from which sex-specific visual anger codes and brain scores were derived. In these analyses, brain scores were significantly associated with emotional symptoms in females and showed associations in the same direction but of smaller magnitude in males (table S4). The direction of effects was consistent across sexes. Moreover, interaction analyses revealed no significant brain score × sex interaction on emotional symptoms, indicating that the observed associations were not driven by sex differences.

### Computational marker predicted the onset of emotional symptoms at age 23 years

**Fig. 5.**
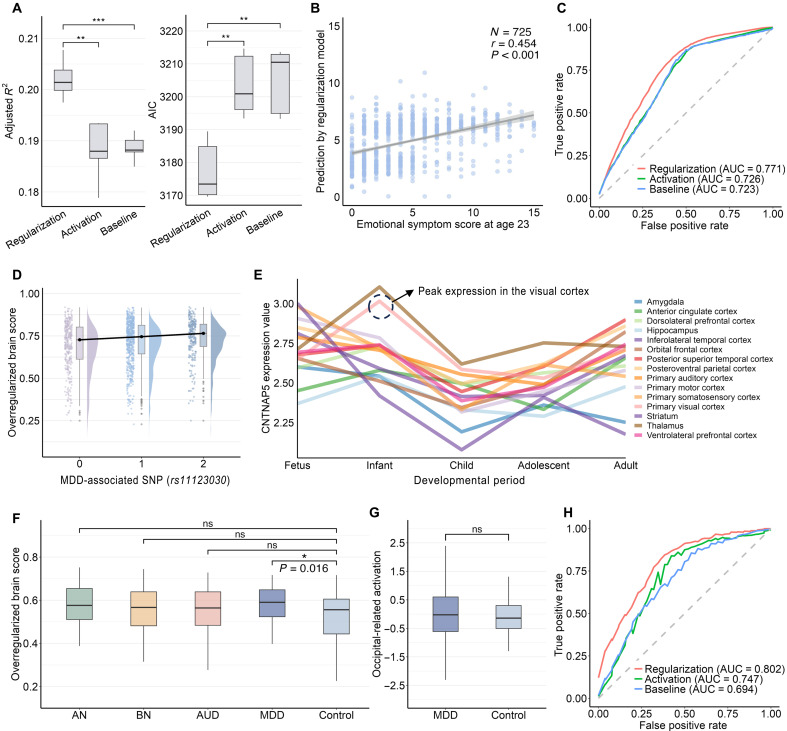
Clinical relevance of the computational marker. (**A**) Comparison of model performance (adjusted *R*^2^ and AIC) across the regularization, activation (using the first PC), and baseline models for predicting emotional symptoms at age 23 years using data from age 19 years among individuals without clinically relevant emotional symptoms at age 19 years. Each model was evaluated using fivefold cross-validation with 100 repetitions. (**B**) Observed emotional symptom scores at age 23 years and predictions from the regularization model. (**C**) Comparison of AUC across the regularization, activation (using the first PC), and baseline models for classifying individuals into high- and low-risk groups among those without clinically relevant emotional symptoms at age 19 years. (**D**) The overregularized brain score was associated with an MDD-related SNP *rs11123030*, which was reported in a recent MDD GWAS (*30*). This SNP is mapped to the *CNTNAP5* gene. (**E**) Expression of *CNTNAP5* across 15 brain regions. (**F**) Case-control comparison of the overregularized brain scores. (**G**) Case-control comparison of the first PC extracted from the traditional brain activation biomarker using the first PC. (**H**) Comparison of AUC across the regularization, activation (using the first PC), and baseline models for classifying individuals into MDD and healthy controls. *P < 0.05; **P < 0.01; ***P < 0.001; ns, nonsignificant.

Among the IMAGEN individuals without clinically relevant emotional symptoms at age 19 years (*N* = 725, 57.24% girls), we found that the regularization model including the computational markers (i.e., the optimal- and overregularized brain scores) at age 19 years could predict emotional symptoms at age 23 years (adjusted $R^{2}=0.202\pm 0.004$; Fig. 5, A and B). This prediction performed significantly better, when compared with both the baseline model (adjusted $R^{2}=0.189\pm 0.003$) and the brain activation model (adjusted $R^{2}=0.188\pm 0.006$). The baseline model used the sex, data collection site, handedness, and socioeconomic status as predictors. The brain activation model additionally included the traditional brain activation biomarker. Last, the regularization model extended the baseline model by adding the optimal- and overregularized brain scores (see Materials and Methods).

Following the literature (*26*), we identified 195 high-risk individuals whose emotional symptoms scored above the 75th percentile in the IMAGEN sample at age 23 years. The regularization model achieved the best accuracy, as measured by the area under the receiver operating characteristic curve (AUC = 0.771 ± 0.029; Fig. 5C), and performed significantly better than the activation and baseline models (∆AUC>0.045;paired t>35.88,P<0.001). To contextualize this performance, we further demonstrated that the computational markers hold predictive value that complements established environmental risk factors like family stress (see Supplementary Text).

### Computational marker associated specifically with depression risk

Next, we examined the associations of the overregularized brain score with depression-related risk genetic variants. To assess specificity, we also tested its associations with genetic risks for other psychiatric disorders characterized by dysfunctional processing of negative emotional stimuli, including alcohol use disorder (AUD) (*27*), anorexia nervosa (AN) (*28*), and bulimia nervosa (BN) (*29*). Among 171 single nucleotide polymorphisms (SNPs) implicated in recent large-scale genome-wide association studies (GWAS) of MDD (*30*), AUD (*31*), AN (*32*), and BN (*33*), we identified a significant association with the risk allele T of *rs11123030* in the IMAGEN cohort (*N* = 1016, β = 0.028, 95% CI = [0.004, 0.042], *P* = 4×10^−6^, false discovery rate (FDR) *q* = 1×10^−4^; Fig. 5D). This SNP has reached genome-wide significance in the MDD GWAS (*30*). As a control condition, when we considered the traditional brain activation biomarker (i.e., the anger minus neutral contrast value of the occipital-related network during the face task), we could not identify any significant genetic association. This SNP *rs11123030* is an intron variant on the gene CNTNAP5 (contactin-associated protein family member 5), which is a member of the neurexin superfamily playing critical roles in neuronal development and axonal guidance (*34*). Using the BrainSpan database (*35*), we found that the gene expression of CNTNAP5 in the primary visual cortex peaked during infancy (Fig. 5E), which might align with the previous reports that 4- and 6-month-old infants can discriminate anger and neutral facial expressions (*36*). Given that psychiatric traits are highly polygenic, we further examined whether the overregularized brain score was associated with polygenic risk scores (PRSs) and found a specific association with PRS_MDD_ (β = 0.07, 95% CI = [0.006, 0.134], *P* = 0.031).

### Computational marker detected specifically in MDD patients

We investigated these computational markers during the same emotional face task in the STRATIFY/ESTRA clinical cohort (ages 19 to 25 years) (*37*). Compared to healthy controls (*N* = 62), we found that patients with MDD (*N* = 134) showed lower optimal-regularized brain scores (β = −0.054, 95% CI = [−0.096, −0.012], *P* = 0.012) and higher overregularized brain scores (β = 0.044, 95% CI = [0.008, 0.081], *P* = 0.016; Fig. 5F). However, such case-control differences were not significant in patients with AUD (*N* = 122), AN (*N* = 52), or BN (*N* = 41). Again, when we considered the traditional brain activation biomarker, we could not detect any significant case-control difference (Fig. 5G). The MDD-control classification model using these computational markers achieved a high accuracy (AUC = 0.802 ± 0.060 given by fivefold cross-validation with 100 repetitions), which outperformed both the activation and baseline classification models (∆AUC > 0.055, paired *t* > 27.547, *P* < 0.001; Fig. 5H).

## DISCUSSION

This study combined a computational perturbation approach with large-scale neuroimaging data to characterize the neurocomputational mechanisms of depression. By analyzing the face-task fMRI data from 1332 adolescents, we linked the weakened neural representations of angry faces in the visual system to emotional symptoms. In the dual-pathway DNN model with optimal parameters, its codes in a latent layer reproduced the neural representations in the visual system while processing angry faces. Perturbation of a mechanistic parameter (i.e., the concept-regularization strength) in the DNN suggested that overreliance on preexisting concepts may act as a pathological mechanism, driving negative bias in emotion perception. In plain terms, “regularization” in our model mimics how the brain uses past knowledge to make sense of what we see. When this process is too strong (“overregularization”), abstract beliefs about anger can overpower the actual visual details of a face. This computationally produces a “negative bias,” where the perception is distorted by expectation rather than driven by the sensory reality. This mechanism enabled the derivation of a computational marker, which predicted high-risk adolescents 4 years before symptom onset, demonstrated genetic association with MDD, and was specifically detectable in MDD patients. Notably, these findings were not observed using traditional brain activation biomarkers. Our results suggest excessive neural regularization as a potential pathological mechanism in depression and provide a predictive computational marker for early prevention and targeted intervention strategies.

Our finding that weakened visual neural representations were associated with emotional symptoms highlights a bottom-up contribution to depression. While previous studies have emphasized top-down biases in appraisal and regulation (*38*), we show that social threat signals are already degraded during the cortical visual processing, constraining subsequent higher-order cognitive processes. This points to a functional role for occipital dysfunction as a sensory coding bottleneck for social threat signals, which helps explain recently accumulating multimodal evidence of visual cortex abnormalities in depression (*39*). Within the Bayesian brain framework (*40*), noisy sensory evidence increases the influence of priors, rendering perception less constrained by external reality but more susceptible to bias, thereby increasing vulnerability to depression. This interpretation is further supported by our findings that both a genetic risk variant for depression (i.e., *rs11123030*) and the PRS for depression were associated with the occipital-derived computational marker, in line with prior work linking polygenic risk for depression to the occipital cortex (*41*).

Our findings suggest a model of offline optimization that embeds priors for fast inference during emotion perception. The theory of constructed emotion (*9*) posits that an instance of emotion is created when sensory data are categorized using emotion concept knowledge (*42*). The construction process is often theorized as a Bayesian inference, which involves computationally expensive online inference (*43*), and its biological plausibility has been under active debate (*44*). While our fMRI data empirically show weakened occipital representations, our dual-pathway DNN model offers a computational analogy to interpret these patterns: Instead of calculating Bayes’ rule in real time, the brain implements its solutions through learned neural structures. Different from standard DNNs for object recognition (*22*), our DNN model is trained “off-line” under regularization by emotion concepts (*16*), forming embedded priors. This process resembles human development, where acquired emotion concepts optimize the neural structures in the visual system to facilitate visual perception for efficiency. After training, there is a rapid, feedforward operation that automatically integrates sensory signals with priors. The biological plausibility of this mechanism is underscored by the similarity between our DNN-derived codes and neural activity, as well as by neuroimaging evidence that emotion schemas are decoded from visual cortex activities during facial emotion processing (*10*).

The findings of the over concept-regularization in the brain’s visual coding for angry faces provided a computational mechanism for the cognitive neuropsychological model of depression (*17*). Different from the traditional cognitive model of depression (*45*), the cognitive neuropsychological model hypothesized that the “bottom-up” negative perceptual biases drive the development of the negative bias in the brain’s information processing, which in turn leads to the “top-down” negative attentional biases (*17*). However, the brain-based and computational mechanism of this hypothesis remained unclear. Here, our perturbation experiment demonstrated that the negative bias in the facial emotion perception increases while the strength of the overregularization increases (Fig. 4B). This mechanism of overreliance on already established priors is supported by the neuroimaging literature reporting the increased functional connectivity between the occipital cortex and the memory system [e.g., the hippocampus (*46*) and the amygdala (*47*)] in patients with depression.

The overregularization in the visual emotion coding for angry faces can be used as a predictive computational marker for depression risk. Neuroimaging studies of depression have identified dysfunctions in many brain regions, including the prefrontal cortex, anterior cingulate, amygdala, and striatum (*48*). However, most neuroimaging biomarkers established from these correlative findings offer limited generalizability to independent samples (*49*). Here, different from these correlative biomarkers, we derived a computational marker from the overregularization mechanism. This marker is predictive to symptom onset and detectable in patients with depression, indicating an early and predictive marker that may gate the development of depression. This marker further suggests the visual cortex as a target of intervention. In a pioneering clinical trial, the repetitive transcranial magnetic stimulation targeting the visual cortex had an antidepressant effect (*50*), which might act through modulating the synapse-associated proteins related to neural plasticity (*51*).

The current study is not without limitations. First, while our main findings in the IMAGEN cohort were generalizable to the independent STRATIFY cohort, these two cohorts mainly cover the white population with middle-to-high socioeconomic status. Therefore, future studies are needed to test whether the main findings could generalize to broader populations with diverse socioeconomic or racial/ethnic backgrounds. Second, the statistically significant differences in emotional symptoms between high- and low-efficiency groups showed small effect sizes (Cohen’s *d* ≈ 0.2). Although this is typical in large-scale, population-based cohorts like IMAGEN, where participants generally exhibit subclinical symptoms rather than severe pathology, this grouping has limited predictive power for individual clinical diagnosis. Third, while our computational marker showed promise in predicting symptom onset and improving classification in the STRATIFY clinical cohort, its broader clinical utility requires further validation in larger, independent clinical cohorts. Future studies should assess the marker’s generalizability across different developmental stages, particularly younger adolescents, given that the steepest increases in depressive symptoms often occur earlier than age 19 years (*52*). In addition, it is crucial to test the marker’s ability to predict symptom persistence versus recovery, which would help distinguish transient distress from chronic pathology and better inform targeted interventions (*53*).

In conclusion, this work proposed a brain-mapped deep learning perturbation approach to probing neurocomputational mechanisms. The findings highlight that the excessive concept regularization in visual anger coding is a predictive computational marker for depression.

## MATERIALS AND METHODS

### Participants

The participants were drawn from the IMAGEN study, a population-based cohort conducted to understand the neurobiological basis of psychological traits and psychiatric disorders in adolescents (*54*). As the IMAGEN cohort is predominantly composed of individuals of Caucasian ancestry, and to enhance the homogeneity and interpretability of the analyses, the present study included only Caucasian participants at age 19 years with available behavioral, clinical, environmental, and functional neuroimaging data. Written informed consent was obtained from all participants and their respective parents or guardians, and the study was approved by the institutional ethics committees of King’s College London (PNM/10/11-126), University of Nottingham (D/11/2007), Trinity College Dublin (SPREC092007-01), Technische Universität Dresden (EK 235092007), Commissariat a l’Energie Atomique et aux Energies Alternatives, INSERM (2007-A00778-45), and University Medical Center at the University of Hamburg (M-191/07) and by the medical ethics committee of the University of Heidelberg (2007-024N-MA) in Germany in accordance with the Declaration of Helsinki.

### Emotional symptoms

Emotional symptoms of the IMAGEN participants were assessed using screening questions from the Strengths and Difficulties Questionnaire (SDQ) (*55*) and the DAWBA (*20*). The SDQ is a valid and reliable assessment commonly used to measure the emotional and behavioral problems in adolescents. The DAWBA is frequently used to characterize clinical symptoms of psychopathology. At age 19 years, the self-reported emotional symptoms included generalized anxiety disorder (7 items), depression (8 items), specific phobia (13 items), and eating disorder (5 items) (*56*), which were selected from SDQ and DAWBA. The choice of using the self-reported version was grounded on the finding that the emotional symptom scores gathered from adolescents themselves are more reliable than those provided by their parents (*57*). The full set of psychiatric questions asked in this study can be found in table S5. Meanwhile, the total score of the ADRS at age 19 years for each participant was used to evaluate adolescent depression (*58*).

### Emotional face task and fMRI preprocessing

The emotional face task was used to elicit activation in the brain’s facial emotion processing system (*59*). In this task, participants passively watched 18-s blocks of either a face movie (presenting faces with angry, happy, or neutral expressions) or a control stimulus (concentric circles). Each face movie showed black-and-white video clips (200 to 500 ms) of male or female faces. Angry face movies depicted faces that started from a neutral expression and dynamically transitioned into an angry expression, whereas neutral face movies displayed ambiguous facial movements without a specific emotional content (e.g., lip licking). Four blocks each of angry, happy, and neutral expressions were interleaved with twelve blocks of control stimulus for participants at age 19 years. In this study, we used the neural reactivity associated with angry expressions to explore the negative emotional processing.

fMRI data were collected at eight research sites with 3 T MRI scanners from various manufacturers (Siemens, Philips, General Electrics, and Bruker). To minimize site-related variability inherent in multisite neuroimaging, we implemented rigorous harmonization procedures at the data acquisition stage. Scanning protocols were specifically standardized to be compatible across all scanners and manufacturers (*54*). In brief, high-resolution T1-weighted structural images were obtained for anatomical localization and co-registration with the functional time series. Blood oxygen level–dependent functional images were acquired using a gradient-echo echoplanar imaging (EPI) sequence. In this task, a hundred and sixty volumes were collected per subject, each containing 40 slices aligned to the anterior-posterior bicommissural line (echo time (TE) = 30 ms, repetition time (TR) = 2200 ms, 2.4-mm slice thickness, 1-mm gap, matrix size of 64 by 64 over a 22 cm–by–22 cm field of view, resulting in a final voxel size of 3.4 mm by 3.4 mm by 3.4 mm).

fMRI data were preprocessed using SPM8 (statistical parametric mapping; www.fil.ion.ucl.ac.uk/spm). Functional time series data were initially corrected for slice timing to adjust for temporal differences, followed by realignment to the first volume. Subsequently, the data were nonlinearly warped on the Montreal Neurological Institute space using a custom EPI template (53 × 63 × 46 voxels). Last, the data were resampled to a resolution of 3 mm by 3 mm by 3 mm and smoothed with a 5-mm full-width at half-maximum isotropic Gaussian kernel.

After fMRI preprocessing, the contrast maps of angry versus neutral faces were used to measure the activation of the brain’s facial emotion processing system responding to angry faces (*N* = 1402). Analyses were confined to gray matter voxels, as defined by the automated anatomical labeling (AAL2) template (47,640 voxels) (*60*). To ensure data quality and exclude participants with unreliable or idiosyncratic activation patterns, we implemented a similarity-based quality control procedure (*56*). Specifically, we computed the interparticipant similarity of activation maps by calculating the mean correlation of each participant’s map with those of all other participants. Participants with mean similarity values falling below the 5th percentile were identified as outliers and excluded (*N* = 70). We confirmed that this approach effectively screened out data affected by head motion, which was quantified using mean FD. The excluded participants exhibited significantly higher mean FD than the retained participants (*t* = 3.47, *P* = 0.0009). This resulted in a final sample of 1332 participants (704 girls) with consistent and reliable activation patterns (fig. S1). Notably, the included sample did not differ significantly from the excluded IMAGEN participants in key demographic or psychological variables, except for head motion (table S6). The voxel-wise activation maps revealed significant group differences in 950, 3139, and 310 voxels across sex, data collection site, and handedness, respectively (after FDR correction, *q* < 0.05). To reduce potential confounding from these factors, we adjusted the activation of each voxel by regressing out the effects of sex, data collection site, and handedness using a regression model, followed by normalization using *z*-scores. These processed activations were then used in the following analyses.

### The analysis of visual anger code

#### VAE model

VAEs work by compressing high-dimensional data into lower-dimensional latent representations, effectively capturing the nonlinear correlations within the data (*61*). VAEs have demonstrated promising potential in extracting neurobiological low-dimensional information from highly entangled neuroimaging data (*18*, *19*). In this study, the VAE model consisted of an encoder network, followed by a latent layer that was passed onto a decoder network of the same size as the encoder layers arranged in reversed order. The model was designed to accommodate a variable number of fully connected hidden layers in both the encoder and decoder networks. Each hidden layer included both batch normalization (*62*) and dropout (*P* = 0.3) (*63*), with Leaky Rectified Linear Units (*64*) as the activation function. The latent layer *z* was set to 256 neurons and was generated by sampling a Gaussian distribution using two fully connected layers of the means μ and standard deviations σ, both with the same size as *z*.

When training the model, the processed activations were vectorized to one input layer and passed through the VAE framework. The model was trained to minimize the following loss function

$$L=L_{\text{recon}}+W_{\text{KLD}}\times \text{KLD}$$

Here, $L_{\text{recon}}$ is the reconstruction error between the input and reconstructed output vectors, calculated using the mean square error. KLD represents the Kullback-Leibler divergence, which penalizes the divergence between the sampling of the latent layer and the prior Gaussian distribution Ν(0,I). $W_{\text{KLD}}$ is a weight put on the KLD, and we set $W_{\text{KLD}}$ = 0.0002 in this study.

The VAE model was implemented using the PyTorch toolbox and trained on a single NVIDIA GeForce RTX 4090 GPU. During training, the Adam optimizer was used with a weight decay of 1 × 10^−5^ and an initial learning rate of 1 × 10^−4^. The learning rate was adjusted using a cosine schedule with a period of 5. The number of training epochs was set to 500 to ensure sufficient training to learn the complexity of the data.

During the inference stage, we masked the processed activations with the occipital-related network, which was derived a priori in our previous work using sparse nonnegative matrix factorization of the brain responses to angry faces (*8*). Activations within this network (14,218 voxels) were retained, and all values outside the network were set to zero, yielding a sparse input vector for each participant. This vector was then fed into the trained VAE model to extract the layer of means (μ) as the visual anger code for each participant.

#### Hyperparameter selection and stability testing

To optimize the VAE model, we evaluated several hyperparameters, including batch sizes (16, 32, and 64); network structures with one hidden layer of 1024 neurons, two hidden layers with 1024 and 512 neurons, and three hidden layers with 1024, 512, and 256 neurons; learning rates (1 × 10^−5^, 1 × 10^−4^, and 1 × 10^−3^); dropout rates (0, 0.1, 0.3, and 0.5); and the dimensionality of the latent layer *z* (128, 256, and 512 neurons). Given the unsupervised nature of the VAE and the computational demands of the model, we used a standard fivefold cross-validation approach for hyperparameter tuning. For each configuration, reconstruction performance was quantified by the cosine similarity between the reconstructed output and the input array on the validation folds. The average cosine similarity across the five folds was calculated to summarize the performance of each configuration. Crucially, this selection was based solely on unsupervised reconstruction metrics, ensuring that the hyperparameter tuning process remained independent of the subsequent statistical analyses involving clinical variables. Next, the model stability was evaluated by repeating training three times with the same hyperparameters but different random seeds. For each participant, we calculated the difference in cosine similarity to all other participants. If the model produced consistent results, the average change in cosine similarity should be zero. The model with the average change closest to zero was considered the most stable. From this analysis, the optimal VAE hyperparameters were found to be a batch size of 64 with two hidden layers (1024 and 512 neurons), a learning rate of 1 × 10^−4^, a dropout rate of 0.3, and a latent dimensionality of 256 (see Supplementary Text and fig. S2). See figs. S3 to S6 for an illustration on the validity of the final VAE model used in this study.

#### Clustering analysis

We used *k*-means clustering to identify distinct patterns of visual anger codes in the VAE latent space. Clustering was performed on the 256-dimensional latent representations, which are regularized by a standard normal prior during VAE training. This prior encourages the latent space to be approximately Euclidean and near-isotropic, making a centroid-based clustering approach well matched to the geometry of the representation. *K*-means clustering was implemented using Euclidean distance, which minimizes the within-cluster sum of squared Euclidean distances between visual anger codes and their assigned centroids. The candidates for optimal number of clusters for the *k*-means approach were determined using the silhouette score, which measures the spread of the clusters, and the elbow method, which uses the sum of squared distances to assess intercluster separation. Next, the final clusters were selected based on a consensus matrix generated by clustering 100 times using the same parameters to ensure cluster stability. In addition, to assess the robustness and reproducibility, clustering stability was also evaluated on a held-out test set (see Supplementary Text). Across these analyses, we identified two subgroups as the optimal number of clusters.

#### Information gain of visual anger code

Information gain is a commonly used metric to quantify the reduction in uncertainty providing the coded information, where the uncertainty is often measured by the entropy in the information code (*65*). Here, the hidden layer of means (μ) in the VAE was used as the 256-dim code vector for human visual anger code. When the values of the 256 elements in the code vector (i.e., the code value) are drawn from a uniform distribution, the entropy (i.e., uncertainty) of the coding reaches its maximum, $H_{\text{uniform}}$. Comparing with the entropy calculated from the distribution of the actual code value ($H_{\text{actual}}$), the information gain was estimated as ${\text{IG}}_{\text{vec}}=H_{\text{uniform}}-H_{\text{actual}}$. This metric corresponds to the KLD from a uniform distribution and quantifies the degree to which the visual anger code is structured versus near-random. Accordingly, a higher ${\text{IG}}_{\text{vec}}$ suggests that the encoding is more informative and less random, whereas a lower ${\text{IG}}_{\text{vec}}$ indicates a representation that is closer to maximal uncertainty (i.e., more random or noisy), reflecting reduced coding efficiency. See Supplementary Text for more details.

To determine whether there was a significant difference in ${\text{IG}}_{\text{vec}}$ between the two groups derived from the clustering analysis, we established the 95% CI of the difference by 1000 bootstraps. For each bootstrap iteration, we performed random sampling with replacement for both groups, with the number of samples equal to the size of each group. We then averaged the visual anger code across participants within each group and calculated the difference in ${\text{IG}}_{\text{vec}}$ between the two resampled groups. This process was repeated 1000 times to construct the 95% CI for the difference. If the 95% CI did not include 0, we considered the difference in ${\text{IG}}_{\text{vec}}$ between the two groups to be statistically significant.

#### Symptom difference

We built a linear regression model to test the association between symptom scores and group, where group, derived from the clustering analysis, was treated as a discrete predictor. Data collection site, sex, handedness, and socioeconomic status were regressed out as covariates in this analysis and the following linear regression models. Socioeconomic status was quantified using the total score from 16 family-stress items of the DAWBA, with each item rated on a three-point scale: 0 (“No, or does not apply”), 1 (“A little”), and 2 (“A lot”). The coefficient (standardized β) of the linear regression models and its 95% CI were reported.

### Mapping

#### Concept-regularized perceptual network

Concept-regularized perceptual network (CRPN) is a brain-inspired network established in our prior study with reduced complexity and enhanced biological interpretability (*16*). In brief, CRPN models the process by which emotion concepts in high-level brain regions shape the perceptual coding of emotional faces. We built the baseline model, named PerceptNet, for visual perception using ResNet18, which is a well-established convolutional neural network for object recognition. The last layer of ResNet18 was replaced by two fully connected layers for feature coding, each with 256 neurons, followed by two fully connected layers with 128 and 7 neurons, respectively, for seven-class facial emotion recognition. We have proposed the CRPN framework in which the feature coding of PerceptNet is regularized by another neural network for emotional concepts, which adapted an orthogonal coding scheme to disentangle emotion-concept and nonemotional-concept features (*16*). During its training process, this regularization is implemented by the following loss function

$$L_{\text{CRPN}}=\lambda_{\text{CE}}L_{\text{CE}}+\lambda_{D}L_{D}+\lambda_{\text{reg}}L_{\text{reg}}$$

where $L_{\text{CE}}$ is the loss related to the emotion recognition task, $L_{D}$ is used to enhance the richness of emotional information in the perceptual coding, and $L_{\text{reg}}$ is the loss associated with emotion concept regularization. The parameters $\lambda_{\text{CE}}$, $\lambda_{D}$, and $\lambda_{\text{reg}}$ control the importance of different constraints, where $\lambda_{\text{reg}}$ controls the regularization strength of emotion concepts on the perceptual coding. After training, the concept-regularized PerceptNet, also noted by CRPN, is used for the inference without referring to the neural network for emotional concepts. On the independent testing dataset with 400 human face expressions for seven emotions, this network achieved the best performance compared with the previous networks in that study (*16*).

#### Construction of CRPN contrast maps

**Fig. 6.**
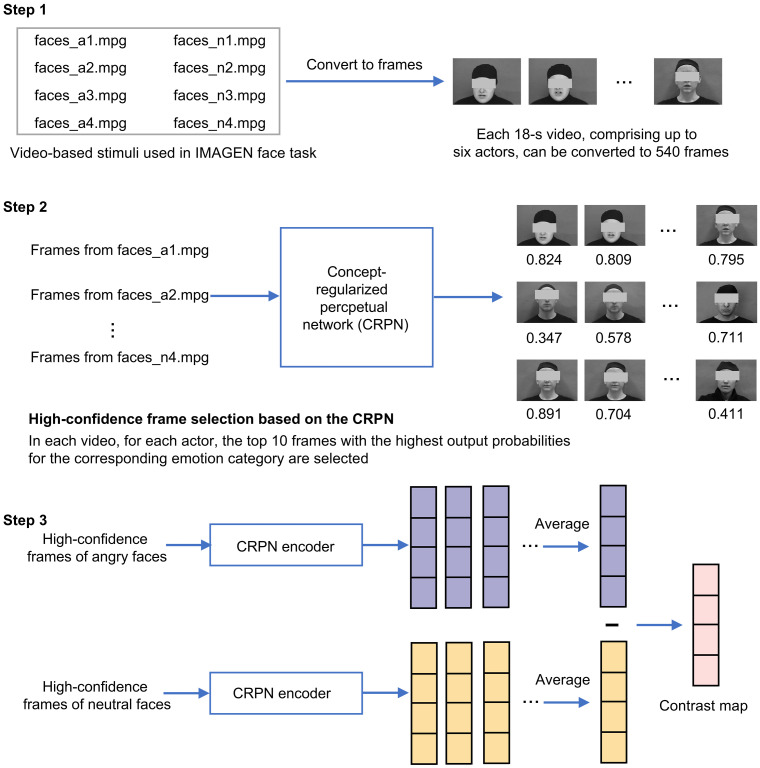
Construction of CRPN contrast maps for angry versus neutral faces. The schematic illustrates the pipeline for constructing CRPN contrast maps using IMAGEN face-task stimuli.

To derive the CRPN anger code, we applied the trained CRPN to the IMAGEN age-19 face-task stimuli (four angry and four neutral 18-s videos, 30 fps, 540 frames per video). Because the videos are composed of sequential single-actor clips, each frame contains only one actor; actor identity and clip boundaries were obtained via manual annotation. Each RGB frame was resized to 224 × 224 and normalized using ImageNet mean and standard deviation, then processed independently (framewise) by the CRPN. For each actor within each video, we selected the top 10 frames with the highest softmax probability for the target emotion category, yielding high-confidence angry and neutral frames. The selected frames were passed through the CRPN encoder to obtain one 256-dimensional embedding per frame, and the contrast code (“CRPN code”) was computed as the difference between the mean angry and mean neutral embeddings (Fig. 6). Full procedural details are provided in Supplementary Text.

#### Brain scores

Because the CRPN code and the brain’s visual anger code lack a one-to-one topological mapping (i.e., dimension *i* in the 256-dimensional CRPN code does not correspond to dimension *i* in the 256-dimensional visual anger code), element-wise metrics like cosine similarity or Pearson correlation are mathematically invalid. Instead, we adopted a distributional approach to evaluate their similarity. First, we averaged the brain’s visual anger codes across participants. Next, we evaluated the mapping between the CRPN code *c* and the brain’s visual anger code *p* using Jensen-Shannon divergence (JS divergence). The mapping procedure was as follows: (i) Both *c* and *p* were normalized to obtain standardized vectors ranging from −1 to 1; (ii) each standardized vector was divided into 40 bins, and the frequency of elements falling within each bin was calculated and converted into probability distributions, resulting in *C* and *P*; (iii) the similarity between the two probability distributions was assessed using JS divergence, with the brain score S(p,c) defined as 1−JS(C∣∣P), ranging from 0 to 1. A higher brain score indicates a greater similarity between these two systems.

To statistically benchmark the performance of our model against PerceptNet, we used a bootstrapping approach with 1000 iterations. In each iteration, we resampled the visual anger codes across participants with replacement to generate a new averaged brain code and estimated the brain scores for both models. Last, we calculated the difference in brain score (∆*S*) between the two models across all iterations to construct a 95% CI for the difference.

#### Noise ceiling

Given the inherent noise in fMRI recordings, we assessed the amount of explainable signal following the previous work (*66*, *67*). To estimate the noise ceiling, we calculated pairwise coding similarities between each participant’s visual anger code and the mean code of the remaining participants—a procedure similar to the brain score computation—and then averaged the results.

#### Voxel-wise exploratory analysis

For each participant, a sphere with a six-voxel radius was centered on every voxel in the brain. The activation patterns within each sphere were fed into the trained VAE model to extract the μ layer as the coding pattern of each voxel. We then averaged these coding patterns across participants to obtain the mean coding pattern for each voxel. Next, we computed the brain score by comparing the similarity between this mean coding pattern and the CRPN code for each voxel. The same process was repeated to compute the brain score for each voxel using the PerceptNet code.

To interpret the function of the voxel-wise brain score patterns, we compared their spatial distributions to the functional anatomy of the human brain using NeuroSynth (www.neurosynth.org/) (*23*), an online platform for meta-analysis of functional neuroimaging literature. Specifically, we sorted all correlation coefficients in descending order and selected the top 20 terms to characterize the underlying functional profile. Similar terms (e.g., “percept” and “perception”) were merged into a base form to avoid redundancy.

### Perturbation analysis of CRPN

Based on the original CRPN framework ($\lambda_{\text{reg}}=1$), we adjusted the $\lambda_{\text{reg}}$ within the range of 0 to 2, with a band of 0.1. For each $\lambda_{\text{reg}}$ value, we trained five instances with the same configurations as in the previous work (*16*), but using different random seeds (i.e., 0, 10, 100, 1000, and 10,000). A higher $\lambda_{\text{reg}}$ indicates increased regularization strength of emotion concepts on perceptual coding. Subsequently, we computed the information gain and negative perceptual bias for CRPN code under different regularization levels.

For each $\lambda_{\text{reg}}$ value, we computed the brain scores between CRPN code and the brain’s visual anger code in both the high-efficiency group and the low-efficiency group. The difference in brain scores between the two groups at each $\lambda_{\text{reg}}$ value is given by

$$\Delta S\left(\lambda_{\text{reg}}\right)=S\left[p_{\text{High}},c\left(\lambda_{\text{reg}}\right)\right]-S\left[p_{\text{Low}},c\left(\lambda_{\text{reg}}\right)\right]$$

We used 1000 bootstraps to establish the 95% CIs for the $\Delta S\left(\lambda_{\text{reg}}\right)$. For each bootstrap iteration, we performed random sampling with replacement for both groups, with the number of samples equal to the size of each group. We then averaged the visual anger code across participants within each group and calculated the $\Delta S\left(\lambda_{\text{reg}}\right)$ in terms of these two resampled groups. This process was repeated 1000 times to construct the 95% CI for the $\Delta S\left(\lambda_{\text{reg}}\right)$. If the 95% CI was greater than 0, the CRPN code more closely resembled the visual anger code of the high-efficiency group; if it was less than 0, it more closely resembled that of the low-efficiency group.

We computed three distinct brain scores for each participant *i*, each corresponding to one of three CRPN conditions: under-regularization, optimal regularization, and overregularization. For each condition *t*, the brain score was computed as follows

$$S_{t,i}=\frac{1}{m}\sum_{j}S\left(p_{i},c_{t,j}\right)$$

where $S\left(p_{i},c_{t,j}\right)$ represents the brain score between the participant’s visual anger code $p_{i}$ and the CRPN code under the *j*-th $\lambda_{\text{reg}}$ value within each regularization condition. Here, *m* denotes the number of $\lambda_{\text{reg}}$ values used for each condition. Next, for each regularization condition, we built a linear regression model to examine the association between the symptom score at age 19 years and the brain score.

### Prediction models in early adulthood

We first assessed whether participants exhibited clinically relevant emotional symptoms at age 19 years using the DAWBA questionnaire. Specifically, for each dimension of the emotional symptoms (i.e., generalized anxiety disorder, depression, specific phobia, and eating disorder), DAWBA provides a probabilistic diagnostic score ranging from 0 to 5: 0 (<0.1% diagnosis probability), 1 (0.5%), 2 (3%), 3 (15%), 4 (50%), and 5 (≥70%). Consistent with the previous work (*56*), a threshold score of 4 (≥50%) was used to identify clinically relevant symptoms within a given dimension. Participants meeting this threshold in any dimension were categorized as having clinically relevant emotional symptoms.

Among individuals without such symptoms at age 19 years, we built linear regression models using data collected at that age to predict emotional symptoms at age 23 years. Model performances were evaluated by fivefold cross-validation with 100 repetitions and compared across the regularization, activation, and baseline models using adjusted *R*^2^ and Akaike Information Criterion (AIC).

We also used linear discriminant analysis (LDA) to classify individuals into high- and low-risk groups. To assess the predictability of regularized brain scores, we used paired *t* tests to evaluate the significance of AUC differences between the regularization model and both the activation and baseline models.

For both the regression models predicting emotional symptoms at age 23 years and the LDA models classifying high- versus low-risk groups, we implemented three types of models. The baseline model used the sex, data collection site, handedness, and socioeconomic status as predictors. Based on this, three variants of the activation model were developed by incorporating (i) the first principal component (PC) of the contrast values across the occipital-related network, (ii) the first and second PCs, and (iii) the mean contrast value. All analyses of the activation model accounted for these three variants. The regularization model extended the baseline model by additionally including both the optimal- and overregularized scores.

### Genetic analysis of the brain scores

DNA purification and genotyping were conducted at the Centre National de Génotypage in Paris. DNA was extracted from ∼10 ml of whole blood collected in BD Vacutainer EDTA tubes (Becton, Dickinson and Company), using the Gentra Puregene Blood Kit (QIAGEN) in accordance with the manufacturer’s protocol. SNPs were excluded from further analyses if they exhibited a call rate < 99%, a minor allele frequency < 1%, or significant deviation from Hardy-Weinberg equilibrium (*P* < 1 × 10^−6^). Individuals were excluded if they had ambiguous sex information or excessive missing genotypes (failure rate > 1%). The principal components analysis of ancestral information was performed, and the first 10 PCs were projected from the founders to other relatives.

Of the 171 SNPs identified in recent GWAS of MDD (*30*), AUD (*31*), AN (*32*), and BN (*33*), 26 overlapped with the SNPs that passed quality control in the IMAGEN cohort. We used linear regression models to examine the association between each SNP and the brain score, controlling for sex, data collection site, handedness, socioeconomic status, and the first 10 PCs of genetic ancestry as covariates. The same analysis was applied to the traditional brain activation biomarker, derived from the anger minus neutral contrast within the occipital-related network during the face task. Three alternative definitions were examined: (i) the first PC of the contrast values across the network, (ii) the second PC, and (iii) the mean contrast value across the network.

We calculated PRSs using PLINK (version 2.0) to capture cumulative genetic risk. For MDD, we used GWAS summary statistics from the Psychiatric Genomics Consortium (135,458 cases and 344,901 controls) as the discovery sample (*68*). For AUD, the discovery sample comprised 121,604 individuals with AUDIT total scores from the UK Biobank (*31*). For AN, the discovery sample consisted of 16,992 cases and 55,525 controls from both Anorexia Nervosa Genetics Initiative and the Eating Disorders Working Group of the Psychiatric Genomics Consortium (*32*). All discovery samples consisted of European-ancestry participants, consistent with the IMAGEN adolescents analyzed in this study. In line with previous work (*56*), the PRSs were computed as the mean across seven *P* value thresholds (0.001, 0.05, 0.10, 0.20, 0.30, 0.40, and 0.50). We did not calculate a PRS for BN because the currently available GWAS for BN (*33*) are based on relatively small sample sizes (151 cases and 2291 controls, only females) and are therefore unsuitable as discovery samples.

### Applicability in a clinical dataset

To evaluate the clinical applicability of regularized brain scores, we used the data from the STRATIFY and ESTRA cohorts. This independent dataset was created using data from two interrelated studies, which recruited participants (ages 19 to 25 years) with MDD or AUD (STRATIFY), AN or BN (ESTRA), and controls with no mental disorder diagnosis at three sites (Berlin, London, and Southampton). The protocol of both studies was harmonized to match the IMAGEN protocol. The contrast map of angry versus neutral faces from the emotional face task for each participant was preprocessed following the same procedures as in the IMAGEN study. Following quality control procedures consistent with those used in the IMAGEN study, we included 134 individuals with MDD, 122 with AUD, 52 with AN, 41 with BN, and 62 healthy controls in further analysis. We applied the trained VAE model again to compute the visual anger code for each participant in the STRATIFY and ESTRA cohorts. Next, we computed the optimal- and overregularized brain scores for each participant. Linear regression models were used to examine the association between each brain score and group (i.e., case or control), with data collection site, sex, and age as covariates. These models also tested differences in the traditional brain activation biomarker between cases and controls. Last, we used LDA to classify individuals into case and control groups.

### Statistical analysis

To compare the demographic variables (sex, handedness, and data collection site) between the high- and low-efficiency groups, we performed χ^2^ test. For socioeconomic status, we used unpaired *t* test instead. To assess whether the coding similarity between the CRPN code and the brain’s visual anger code was statistically significant, we used a spin test. Specifically, we generated a null distribution by rotating the occipital-related activation patterns 1000 times while preserving their spatial structure. For each rotation, we computed the brain score between the CRPN code and the visual anger code derived from the rotated pattern. This procedure yielded a null distribution of brain scores. Then, the true brain score was compared with this distribution to determine a *P* value of rejecting the null distribution. Analysis of variance (ANOVA) was performed to test for significant differences in the information gain of the CRPN code across varying levels of regularization strength $\lambda_{\text{reg}}$.

## Supplementary Materials

### This PDF file includes:

List of IMAGEN Consortium members List of STRATIFY/ESTRA Consortium members Supplementary Text Figs. S1 to S15 Tables S1 to S6

## References

[1] GBD 2021 Diseases and Injuries Collaborators, Global incidence, prevalence, years lived with disability (YLDs), disability-adjusted life-years (DALYs), and healthy life expectancy (HALE) for 371 diseases and injuries in 204 countries and territories and 811 subnational locations, 1990-2021: A systematic analysis for the Global Burden of Disease Study 2021. Lancet 403, 2133–2161 (2024). 38642570 10.1016/S0140-6736(24)00757-8 PMC11122111

[2] R. S. McIntyre, M. Alsuwaidan, B. T. Baune, M. Berk, K. Demyttenaere, J. F. Goldberg, P. Gorwood, R. Ho, S. Kasper, S. H. Kennedy, J. Ly-Uson, R. B. Mansur, R. H. McAllister-Williams, J. W. Murrough, C. B. Nemeroff, A. A. Nierenberg, J. D. Rosenblat, G. Sanacora, A. F. Schatzberg, R. Shelton, S. M. Stahl, M. H. Trivedi, E. Vieta, M. Vinberg, N. Williams, A. H. Young, M. Maj, Treatment-resistant depression: Definition, prevalence, detection, management, and investigational interventions. World Psychiatry 22, 394–412 (2023). 37713549 10.1002/wps.21120 PMC10503923

[3] A. Forte, R. J. Baldessarini, L. Tondo, G. H. Vázquez, M. Pompili, P. Girardi, Long-term morbidity in bipolar-I, bipolar-II, and unipolar major depressive disorders. J. Affect. Disord. 178, 71–78 (2015). 25797049 10.1016/j.jad.2015.02.011

[4] V. Patel, S. Saxena, C. Lund, G. Thornicroft, F. Baingana, P. Bolton, D. Chisholm, P. Y. Collins, J. L. Cooper, J. Eaton, H. Herrman, M. M. Herzallah, Y. Huang, M. J. D. Jordans, A. Kleinman, M. E. Medina-Mora, E. Morgan, U. Niaz, O. Omigbodun, M. Prince, A. Rahman, B. Saraceno, B. K. Sarkar, M. De Silva, I. Singh, D. J. Stein, C. Sunkel, J. UnÜtzer, The *Lancet* Commission on global mental health and sustainable development. Lancet 392, 1553–1598 (2018). 30314863 10.1016/S0140-6736(18)31612-X

[5] A. Stuhrmann, T. Suslow, U. Dannlowski, Facial emotion processing in major depression: A systematic review of neuroimaging findings. Biol. Mood Anxiety Disord. 1, 10 (2011). 22738433 10.1186/2045-5380-1-10 PMC3384264

[6] L. N. Yatham, Biomarkers for clinical use in psychiatry: Where are we and will we ever get there? World Psychiatry 22, 263–264 (2023). 37159356 10.1002/wps.21079 PMC10168147

[7] L. Collin, J. Bindra, M. Raju, C. Gillberg, H. Minnis, Facial emotion recognition in child psychiatry: A systematic review. Res. Dev. Disabil. 34, 1505–1520 (2013). 23475001 10.1016/j.ridd.2013.01.008

[8] H. Lu, E. T. Rolls, H. Liu, D. J. Stein, B. J. Sahakian, R. Elliott, T. Jia, C. Xie, S. Xiang, N. Wang, T. Banaschewski, A. L. W. Bokde, S. Desrivières, H. Flor, A. Grigis, H. Garavan, A. Heinz, R. Brühl, J. L. Martinot, M. P. Martinot, E. Artiges, F. Nees, D. P. Orfanos, H. Lemaitre, L. Poustka, S. Hohmann, N. Holz, J. H. Fröhner, M. N. Smolka, N. Vaidya, H. Walter, R. Whelan, G. Schumann, J. Feng, Q. Luo, Genetic risk-dependent brain markers of resilience to childhood trauma. Nat. Commun. 16, 6219 (2025). 40617820 10.1038/s41467-025-61471-0 PMC12228777

[9] L. F. Barrett, The theory of constructed emotion: an active inference account of interoception and categorization. Soc. Cogn. Affect. Neurosci. 12, 1833 (2017). 28472391 10.1093/scan/nsx060 PMC5691871

[10] P. A. Kragel, M. C. Reddan, K. S. LaBar, T. D. Wager, Emotion schemas are embedded in the human visual system. Sci. Adv. 5, eaaw4358 (2019). 31355334 10.1126/sciadv.aaw4358 PMC6656543

[11] J. A. Brooks, J. Chikazoe, N. Sadato, J. B. Freeman, The neural representation of facial-emotion categories reflects conceptual structure. Proc. Natl. Acad. Sci. U.S.A. 116, 15861–15870 (2019). 31332015 10.1073/pnas.1816408116 PMC6689944

[12] M. C. Camacho, A. N. Nielsen, D. Balser, E. Furtado, D. C. Steinberger, L. Fruchtman, J. P. Culver, C. M. Sylvester, D. M. Barch, Large-scale encoding of emotion concepts becomes increasingly similar between individuals from childhood to adolescence. Nat. Neurosci. 26, 1256–1266 (2023). 37291338 10.1038/s41593-023-01358-9 PMC12045037

[13] N. L. Colich, L. C. Foland-Ross, C. Eggleston, M. K. Singh, I. H. Gotlib, Neural aspects of inhibition following emotional primes in depressed adolescents. J. Clin. Child Adolesc. Psychol. 45, 21–30 (2016). 25635920 10.1080/15374416.2014.982281 PMC4520793

[14] N. L. Colich, T. C. Ho, L. C. Foland-Ross, C. Eggleston, S. J. Ordaz, M. K. Singh, I. H. Gotlib, Hyperactivation in cognitive control and visual attention brain regions during emotional interference in adolescent depression. Biol. Psychiatry Cogn. Neurosci. Neuroimaging 2, 388–395 (2017). 28890942 10.1016/j.bpsc.2016.09.001 PMC5586219

[15] Y. Jiang, A theory of the neural mechanisms underlying negative cognitive bias in major depression. Front. Psych. 15, 1348474 (2024). 10.3389/fpsyt.2024.1348474 PMC10963437 38532986

[16] H. Lu, X. Zhuang, Q. Luo, A brain-inspired way of reducing the network complexity via concept-regularized coding for emotion recognition. Proc. AAAI Conf. Artif. Intell. 38, 556–564 (2024).

[17] J. P. Roiser, R. Elliott, B. J. Sahakian, Cognitive mechanisms of treatment in depression. Neuropsychopharmacology 37, 117–136 (2012). 21976044 10.1038/npp.2011.183 PMC3238070

[18] N. Qiang, Q. Dong, Y. Sun, B. Ge, T. Liu, “Deep variational autoencoder for modeling functional brain networks and ADHD identification” in *2020 IEEE 17th International Symposium on Biomedical Imaging (ISBI)* (IEEE, 2020), pp. 554–557.

[19] X. Zhang, E. A. Maltbie, S. D. Keilholz, Spatiotemporal trajectories in resting-state FMRI revealed by convolutional variational autoencoder. Neuroimage 244, 118588 (2021). 34607021 10.1016/j.neuroimage.2021.118588 PMC8637345

[20] R. Goodman, T. Ford, H. Richards, R. Gatward, H. Meltzer, The Development and Well-Being Assessment: Description and initial validation of an integrated assessment of child and adolescent psychopathology. J. Child Psychol. Psychiatry 41, 645–655 (2000). 10946756

[21] M. Schrimpf, J. Kubilius, H. Hong, N. J. Majaj, R. Rajalingham, E. B. Issa, K. Kar, P. Bashivan, J. Prescott-Roy, F. Geiger, Brain-score: Which artificial neural network for object recognition is most brain-like? bioRxiv 407007 (2018) [cited 2018]. 10.1101/407007.

[22] C. Zhuang, S. Yan, A. Nayebi, M. Schrimpf, M. C. Frank, J. J. DiCarlo, D. L. K. Yamins, Unsupervised neural network models of the ventral visual stream. Proc. Natl. Acad. Sci. U.S.A. 118, e2014196118 (2021). 33431673 10.1073/pnas.2014196118 PMC7826371

[23] T. Yarkoni, R. A. Poldrack, T. E. Nichols, D. C. Van Essen, T. D. Wager, Large-scale automated synthesis of human functional neuroimaging data. Nat. Methods 8, 665–670 (2011). 21706013 10.1038/nmeth.1635 PMC3146590

[24] T. Leist, M. R. Dadds, Adolescents’ ability to read different emotional faces relates to their history of maltreatment and type of psychopathology. Clin. Child Psychol. Psychiatry 14, 237–250 (2009). 19293321 10.1177/1359104508100887

[25] P. Bakan, Extraversion-introversion and improvement in an auditory vigilance task. Br. J. Psychol. 50, 325–332 (1959). 13795958 10.1111/j.2044-8295.1959.tb00711.x

[26] E. D. Hill, P. Kashyap, E. Raffanello, Y. Wang, T. E. Moffitt, A. Caspi, M. Engelhard, J. Posner, Prediction of mental health risk in adolescents. Nat. Med. 31, 1840–1846 (2025). 40044931 10.1038/s41591-025-03560-7 PMC12176513

[27] C. R. Freeman, C. E. Wiers, M. E. Sloan, A. Zehra, V. Ramirez, G. J. Wang, N. D. Volkow, Emotion recognition biases in alcohol use disorder. Alcohol. Clin. Exp. Res. 42, 1541–1547 (2018). 10.1111/acer.13802 29975424

[28] A. Harrison, K. Tchanturia, J. Treasure, Attentional bias, emotion recognition, and emotion regulation in anorexia: state or trait? Biol. Psychiatry 68, 755–761 (2010). 20591417 10.1016/j.biopsych.2010.04.037

[29] T. Legenbauer, S. Vocks, H. Rüddel, Emotion recognition, emotional awareness and cognitive bias in individuals with bulimia nervosa. J. Clin. Psychol. 64, 687–702 (2008). 18473338 10.1002/jclp.20483

[30] A. Dahl, M. Thompson, U. An, M. Krebs, V. Appadurai, R. Border, S. A. Bacanu, T. Werge, J. Flint, A. J. Schork, S. Sankararaman, K. S. Kendler, N. Cai, Phenotype integration improves power and preserves specificity in biobank-based genetic studies of major depressive disorder. Nat. Genet. 55, 2082–2093 (2023). 37985818 10.1038/s41588-023-01559-9 PMC10703686

[31] S. Sanchez-Roige, A. A. Palmer, P. Fontanillas, S. L. Elson, 23andMe Research Team, the Substance Use Disorder Working Group of the Psychiatric Genomics Consortium, M. J. Adams, D. M. Howard, H. J. Edenberg, G. Davies, R. C. Crist, I. J. Deary, A. M. McIntosh, T. K. Clarke, Genome-wide association study meta-analysis of the Alcohol Use Disorders Identification Test (AUDIT) in two population-based cohorts. Am. J. Psychiatry 176, 107–118 (2019). 30336701 10.1176/appi.ajp.2018.18040369 PMC6365681

[32] H. J. Watson, Z. Yilmaz, L. M. Thornton, C. Hübel, J. R. I. Coleman, H. A. Gaspar, J. Bryois, A. Hinney, V. M. Leppä, M. Mattheisen, S. E. Medland, S. Ripke, S. Yao, P. Giusti-Rodríguez, Anorexia Nervosa Genetics Initiative, K. B. Hanscombe, K. L. Purves, Eating Disorders Working Group of the Psychiatric Genomics Consortium, R. A. H. Adan, L. Alfredsson, T. Ando, O. A. Andreassen, J. H. Baker, W. H. Berrettini, I. Boehm, C. Boni, V. B. Perica, K. Buehren, R. Burghardt, M. Cassina, S. Cichon, M. Clementi, R. D. Cone, P. Courtet, S. Crow, J. J. Crowley, U. N. Danner, O. S. P. Davis, M. de Zwaan, G. Dedoussis, D. Degortes, J. E. DeSocio, D. M. Dick, D. Dikeos, C. Dina, M. Dmitrzak-Weglarz, E. Docampo, L. E. Duncan, K. Egberts, S. Ehrlich, G. Escaramís, T. Esko, X. Estivill, A. Farmer, A. Favaro, F. Fernández-Aranda, M. M. Fichter, K. Fischer, M. Föcker, L. Foretova, A. J. Forstner, M. Forzan, C. S. Franklin, S. Gallinger, I. Giegling, J. Giuranna, F. Gonidakis, P. Gorwood, M. G. Mayora, S. Guillaume, Y. Guo, H. Hakonarson, K. Hatzikotoulas, J. Hauser, J. Hebebrand, S. G. Helder, S. Herms, B. Herpertz-Dahlmann, W. Herzog, L. M. Huckins, J. I. Hudson, H. Imgart, H. Inoko, V. Janout, S. Jiménez-Murcia, A. Julià, G. Kalsi, D. Kaminská, J. Kaprio, L. Karhunen, A. Karwautz, M. J. H. Kas, J. L. Kennedy, A. Keski-Rahkonen, K. Kiezebrink, Y. R. Kim, L. Klareskog, K. L. Klump, G. P. S. Knudsen, M. C. La Via, S. Le Hellard, R. D. Levitan, D. Li, L. Lilenfeld, B. D. Lin, J. Lissowska, J. Luykx, P. J. Magistretti, M. Maj, K. Mannik, S. Marsal, C. R. Marshall, M. Mattingsdal, S. McDevitt, P. McGuffin, A. Metspalu, I. Meulenbelt, N. Micali, K. Mitchell, A. M. Monteleone, P. Monteleone, M. A. Munn-Chernoff, B. Nacmias, M. Navratilova, I. Ntalla, J. K. O’Toole, R. A. Ophoff, L. Padyukov, A. Palotie, J. Pantel, H. Papezova, D. Pinto, R. Rabionet, A. Raevuori, N. Ramoz, T. Reichborn-Kjennerud, V. Ricca, S. Ripatti, F. Ritschel, M. Roberts, A. Rotondo, D. Rujescu, F. Rybakowski, P. Santonastaso, A. Scherag, S. W. Scherer, U. Schmidt, N. J. Schork, A. Schosser, J. Seitz, L. Slachtova, P. E. Slagboom, M. C. T. Slof-Op ‘t Landt, A. Slopien, S. Sorbi, B. Świątkowska, J. P. Szatkiewicz, I. Tachmazidou, E. Tenconi, A. Tortorella, F. Tozzi, J. Treasure, A. Tsitsika, M. Tyszkiewicz-Nwafor, K. Tziouvas, A. A. van Elburg, E. F. van Furth, G. Wagner, E. Walton, E. Widen, E. Zeggini, S. Zerwas, S. Zipfel, A. W. Bergen, J. M. Boden, H. Brandt, S. Crawford, K. A. Halmi, L. J. Horwood, C. Johnson, A. S. Kaplan, W. H. Kaye, J. E. Mitchell, C. M. Olsen, J. F. Pearson, N. L. Pedersen, M. Strober, T. Werge, D. C. Whiteman, D. B. Woodside, G. D. Stuber, S. Gordon, J. Grove, A. K. Henders, A. Juréus, K. M. Kirk, J. T. Larsen, R. Parker, L. Petersen, J. Jordan, M. Kennedy, G. W. Montgomery, T. D. Wade, A. Birgegård, P. Lichtenstein, C. Norring, M. Landén, N. G. Martin, P. B. Mortensen, P. F. Sullivan, G. Breen, C. M. Bulik, Genome-wide association study identifies eight risk loci and implicates metabo-psychiatric origins for anorexia nervosa. Nat. Genet. 51, 1207–1214 (2019).31308545

[33] T. D. Wade, S. Gordon, S. Medland, C. M. Bulik, A. C. Heath, G. W. Montgomery, N. G. Martin, Genetic variants associated with disordered eating. Int. J. Eat. Disord. 46, 594–608 (2013). 23568457 10.1002/eat.22133 PMC3775874

[34] S. Chakraborty, J. Sarma, S. S. Roy, S. Mitra, S. Bagchi, S. Das, S. Saha, S. Mahapatra, S. Bhattacharjee, M. Maulik, M. Acharya, Post-GWAS functional analyses of CNTNAP5 suggests its role in glaucomatous neurodegeneration. bioRxiv 583830 [Preprint] (2024) [cited 2014]. 10.1101/2024.03.14.583830. PMC11651621 39637236

[35] J. A. Miller, S. L. Ding, S. M. Sunkin, K. A. Smith, L. Ng, A. Szafer, A. Ebbert, Z. L. Riley, J. J. Royall, K. Aiona, J. M. Arnold, C. Bennet, D. Bertagnolli, K. Brouner, S. Butler, S. Caldejon, A. Carey, C. Cuhaciyan, R. A. Dalley, N. Dee, T. A. Dolbeare, B. A. Facer, D. Feng, T. P. Fliss, G. Gee, J. Goldy, L. Gourley, B. W. Gregor, G. Gu, R. E. Howard, J. M. Jochim, C. L. Kuan, C. Lau, C. K. Lee, F. Lee, T. A. Lemon, P. Lesnar, B. McMurray, N. Mastan, N. Mosqueda, T. Naluai-Cecchini, N. K. Ngo, J. Nyhus, A. Oldre, E. Olson, J. Parente, P. D. Parker, S. E. Parry, A. Stevens, M. Pletikos, M. Reding, K. Roll, D. Sandman, M. Sarreal, S. Shapouri, N. V. Shapovalova, E. H. Shen, N. Sjoquist, C. R. Slaughterbeck, M. Smith, A. J. Sodt, D. Williams, L. Zöllei, B. Fischl, M. B. Gerstein, D. H. Geschwind, I. A. Glass, M. J. Hawrylycz, R. F. Hevner, H. Huang, A. R. Jones, J. A. Knowles, P. Levitt, J. W. Phillips, N. Sestan, P. Wohnoutka, C. Dang, A. Bernard, J. G. Hohmann, E. S. Lein, Transcriptional landscape of the prenatal human brain. Nature 508, 199–206 (2014). 24695229 10.1038/nature13185 PMC4105188

[36] J. D. LaBarbera, C. E. Izard, P. Vietze, S. A. Parisi, Four- and six-month-old infants’ visual responses to joy, anger, and neutral expressions. Child Dev. 47, 535–538 (1976). 1269322

[37] E. B. Quinlan, T. Banaschewski, G. J. Barker, A. L. W. Bokde, U. Bromberg, C. Büchel, S. Desrivières, H. Flor, V. Frouin, H. Garavan, A. Heinz, R. Brühl, J. L. Martinot, M. L. Paillère Martinot, F. Nees, D. P. Orfanos, T. Paus, L. Poustka, S. Hohmann, M. N. Smolka, J. H. Fröhner, H. Walter, R. Whelan, G. Schumann, IMAGEN Consortium, Identifying biological markers for improved precision medicine in psychiatry. Mol. Psychiatry 25, 243–253 (2020). 31676814 10.1038/s41380-019-0555-5 PMC6978138

[38] J. Joormann, M. E. Quinn, Cognitive processes and emotion regulation in depression. Depress. Anxiety 31, 308–315 (2014). 24668779 10.1002/da.22264

[39] F. Wu, Q. Lu, Y. Kong, Z. Zhang, A comprehensive overview of the role of visual cortex malfunction in depressive disorders: Opportunities and challenges. Neurosci. Bull. 39, 1426–1438 (2023). 36995569 10.1007/s12264-023-01052-7 PMC10062279

[40] K. Friston, The history of the future of the Bayesian brain. Neuroimage 62, 1230–1233 (2012). 22023743 10.1016/j.neuroimage.2011.10.004 PMC3480649

[41] A. E. Miles, F. C. Dos Santos, E. M. Byrne, M. E. Renteria, A. M. McIntosh, M. J. Adams, G. Pistis, E. Castelao, M. Preisig, B. T. Baune, K. O. Schubert, C. M. Lewis, L. A. Jones, I. Jones, R. Uher, J. W. Smoller, R. H. Perlis, D. F. Levinson, J. B. Potash, M. M. Weissman, J. Shi, G. Lewis, B. Penninx, D. I. Boomsma, S. P. Hamilton, Major Depressive Disorder Working Group of the Psychiatric Genomics Consortium, E. Sibille, A. R. Hariri, Y. S. Nikolova, Transcriptome-based polygenic score links depression-related corticolimbic gene expression changes to sex-specific brain morphology and depression risk. Neuropsychopharmacology 46, 2304–2311 (2021). 34588609 10.1038/s41386-021-01189-x PMC8580972

[42] A. Ishai, C. F. Schmidt, P. Boesiger, Face perception is mediated by a distributed cortical network. Brain Res. Bull. 67, 87–93 (2005). 16140166 10.1016/j.brainresbull.2005.05.027

[43] T. Parr, G. Rees, K. J. Friston, Computational neuropsychology and bayesian inference. Front. Hum. Neurosci. 12, 61 (2018). 29527157 10.3389/fnhum.2018.00061 PMC5829460

[44] M. Mangalam, The myth of the Bayesian brain. Eur. J. Appl. Physiol. 125, 2643–2677 (2025). 40569419 10.1007/s00421-025-05855-6 PMC12479598

[45] A. T. Beck, *Cognitive Therapy and the Emotional Disorders* (International Universities Press, 1976).

[46] C. Teng, J. Zhou, H. Ma, Y. Tan, X. Wu, C. Guan, H. Qiao, J. Li, Y. Zhong, C. Wang, N. Zhang, Abnormal resting state activity of left middle occipital gyrus and its functional connectivity in female patients with major depressive disorder. BMC Psychiatry 18, 370 (2018). 30477561 10.1186/s12888-018-1955-9 PMC6258168

[47] E. McGlade, J. Rogowska, J. DiMuzio, E. Bueler, C. Sheth, M. Legarreta, D. Yurgelun-Todd, Neurobiological evidence of sexual dimorphism in limbic circuitry of US veterans. J. Affect. Disord. 274, 1091–1101 (2020). 32663937 10.1016/j.jad.2020.05.016

[48] S. G. Disner, C. G. Beevers, E. A. Haigh, A. T. Beck, Neural mechanisms of the cognitive model of depression. Nat. Rev. Neurosci. 12, 467–477 (2011). 21731066 10.1038/nrn3027

[49] A. Abi-Dargham, S. J. Moeller, F. Ali, C. DeLorenzo, K. Domschke, G. Horga, A. Jutla, R. Kotov, M. P. Paulus, J. M. Rubio, G. Sanacora, J. Veenstra-VanderWeele, J. H. Krystal, Candidate biomarkers in psychiatric disorders: State of the field. World Psychiatry 22, 236–262 (2023). 37159365 10.1002/wps.21078 PMC10168176

[50] Z. Zhang, H. Zhang, C. M. Xie, M. Zhang, Y. Shi, R. Song, X. Lu, H. Zhang, K. Li, B. Wang, Y. Yang, X. Li, J. Zhu, Y. Zhao, T.-F. Yuan, G. Northoff, Task-related functional magnetic resonance imaging-based neuronavigation for the treatment of depression by individualized repetitive transcranial magnetic stimulation of the visual cortex. Sci. China Life Sci. 64, 96–106 (2021). 32542515 10.1007/s11427-020-1730-5

[51] J. Liu, H. Guo, J. Yang, Y. Xiao, A. Cai, T. Zhao, F. Y. Womer, P. Zhao, J. Zheng, X. Zhang, J. Wang, R. Zhu, F. Wang, Visual cortex repetitive transcranial magnetic stimulation (rTMS) reversing neurodevelopmental impairments in adolescents with major psychiatric disorders (MPDs): A cross-species translational study. CNS Neurosci. Ther. 30, e14427 (2024). 37721197 10.1111/cns.14427 PMC10915985

[52] A. S. F. Kwong, D. Manley, N. J. Timpson, R. M. Pearson, J. Heron, H. Sallis, E. Stergiakouli, O. S. P. Davis, G. Leckie, Identifying critical points of trajectories of depressive symptoms from childhood to young adulthood. J. Youth Adolesc. 48, 815–827 (2019). 30671716 10.1007/s10964-018-0976-5 PMC6441403

[53] A. Thapar, S. Collishaw, D. S. Pine, A. K. Thapar, Depression in adolescence. Lancet 379, 1056–1067 (2012). 22305766 10.1016/S0140-6736(11)60871-4 PMC3488279

[54] G. Schumann, E. Loth, T. Banaschewski, A. Barbot, G. Barker, C. Büchel, P. J. Conrod, J. W. Dalley, H. Flor, J. Gallinat, H. Garavan, A. Heinz, B. Itterman, M. Lathrop, C. Mallik, K. Mann, J. L. Martinot, T. Paus, J. B. Poline, T. W. Robbins, M. Rietschel, L. Reed, M. Smolka, R. Spanagel, C. Speiser, D. N. Stephens, A. Ströhle, M. Struve, IMAGEN consortium, The IMAGEN study: Reinforcement-related behaviour in normal brain function and psychopathology. Mol. Psychiatry 15, 1128–1139 (2010). 21102431 10.1038/mp.2010.4

[55] R. Goodman, The Strengths and Difficulties Questionnaire: A research note. J. Child Psychol. Psychiatry 38, 581–586 (1997). 9255702 10.1111/j.1469-7610.1997.tb01545.x

[56] C. Xie, S. Xiang, C. Shen, X. Peng, J. Kang, Y. Li, W. Cheng, S. He, M. Bobou, M. J. Broulidakis, B. M. van Noort, Z. Zhang, L. Robinson, N. Vaidya, J. Winterer, Y. Zhang, S. King, T. Banaschewski, G. J. Barker, A. L. W. Bokde, U. Bromberg, C. Büchel, H. Flor, A. Grigis, H. Garavan, P. Gowland, A. Heinz, B. Ittermann, H. Lemaître, J. L. Martinot, M. P. Martinot, F. Nees, D. P. Orfanos, T. Paus, L. Poustka, J. H. Fröhner, U. Schmidt, J. Sinclair, M. N. Smolka, A. Stringaris, H. Walter, R. Whelan, S. Desrivières, B. J. Sahakian, T. W. Robbins, G. Schumann, T. Jia, J. Feng, IMAGEN Consortium, STRATIFY/ESTRA Consortium, ZIB Consortium, A shared neural basis underlying psychiatric comorbidity. Nat. Med. 29, 1232–1242 (2023). 37095248 10.1038/s41591-023-02317-4 PMC10202801

[57] A. Becker, N. Hagenberg, V. Roessner, W. Woerner, A. Rothenberger, Evaluation of the self-reported SDQ in a clinical setting: do self-reports tell us more than ratings by adult informants? Eur. Child Adolesc. Psychiatry 13, Ii17–Ii24 (2004). 15243782 10.1007/s00787-004-2004-4

[58] A. Revah-Levy, B. Birmaher, I. Gasquet, B. Falissard, The Adolescent Depression Rating Scale (ADRS): A validation study. BMC Psychiatry 7, 2 (2007). 17222346 10.1186/1471-244X-7-2 PMC1785378

[59] M. H. Grosbras, T. Paus, Brain networks involved in viewing angry hands or faces. Cereb. Cortex 16, 1087–1096 (2006). 16221928 10.1093/cercor/bhj050

[60] E. T. Rolls, M. Joliot, N. Tzourio-Mazoyer, Implementation of a new parcellation of the orbitofrontal cortex in the automated anatomical labeling atlas. Neuroimage 122, 1–5 (2015). 26241684 10.1016/j.neuroimage.2015.07.075

[61] D. P. Kingma, M. Welling, Auto-encoding variational Bayes. arXiv:1312.6114v10 [stat.ML] (2013).

[62] S. Ioffe, C. Szegedy, Batch normalization: Accelerating deep network training by reducing internal covariate shift. Proc. ICML 37, 448–456 (2015).

[63] G. E. Hinton, N. Srivastava, A. Krizhevsky, I. Sutskever, R. Salakhutdinov, Improving neural networks by preventing co-adaptation of feature detectors. arXiv:1207.0580 [cs.NE] (2012).

[64] A. L. Maas, A. Y. Hannun, A. Y. Ng, Rectifier nonlinearities improve neural network acoustic models. Proc. ICML 30, 3 (2013).

[65] J. R. Quinlan, Induction of decision trees. Mach. Learn. 1, 81–106 (1986).

[66] A. Shoham, I. D. Grosbard, O. Patashnik, D. Cohen-Or, G. Yovel, Using deep neural networks to disentangle visual and semantic information in human perception and memory. Nat. Hum. Behav. 8, 702–717 (2024). 38332339 10.1038/s41562-024-01816-9

[67] C. Caucheteux, A. Gramfort, J. R. King, Evidence of a predictive coding hierarchy in the human brain listening to speech. Nat. Hum. Behav. 7, 430–441 (2023). 36864133 10.1038/s41562-022-01516-2 PMC10038805

[68] N. R. Wray, S. Ripke, M. Mattheisen, M. Trzaskowski, E. M. Byrne, A. Abdellaoui, M. J. Adams, E. Agerbo, T. M. Air, T. M. Andlauer, S.-A. Bacanu, M. Bækvad-Hansen, A. F. T. Beekman, T. B. Bigdeli, E. B. Binder, D. R. H. Blackwood, J. Bryois, H. N. Buttenschøn, J. Bybjerg-Grauholm, N. Cai, E. Castelao, J. H. Christensen, T.-K. Clarke, J. I. R. Coleman, L. Colodro-Conde, B. Couvy-Duchesne, N. Craddock, G. E. Crawford, C. A. Crowley, H. S. Dashti, G. Davies, I. J. Deary, F. Degenhardt, E. M. Derks, N. Direk, C. V. Dolan, E. C. Dunn, T. C. Eley, N. Eriksson, V. Escott-Price, F. H. F. Kiadeh, H. K. Finucane, A. J. Forstner, J. Frank, H. A. Gaspar, M. Gill, P. Giusti-Rodríguez, F. S. Goes, S. D. Gordon, J. Grove, L. S. Hall, E. Hannon, C. S. Hansen, T. F. Hansen, S. Herms, I. B. Hickie, P. Hoffmann, G. Homuth, C. Horn, J.-J. Hottenga, D. M. Hougaard, M. Hu, C. L. Hyde, M. Ising, R. Jansen, F. Jin, E. Jorgenson, J. A. Knowles, I. S. Kohane, J. Kraft, W. W. Kretzschmar, J. Krogh, Z. Kutalik, J. M. Lane, Y. Li, Y. Li, P. A. Lind, X. Liu, L. Lu, D. J. Mac Intyre, D. F. Mac Kinnon, R. M. Maier, W. Maier, J. Marchini, H. Mbarek, P. M. Grath, P. M. Guffin, S. E. Medland, D. Mehta, C. M. Middeldorp, E. Mihailov, Y. Milaneschi, L. Milani, J. Mill, F. M. Mondimore, G. W. Montgomery, S. Mostafavi, N. Mullins, M. Nauck, B. Ng, M. G. Nivard, D. R. Nyholt, P. F. O’Reilly, H. Oskarsson, M. J. Owen, J. N. Painter, C. B. Pedersen, M. G. Pedersen, R. E. Peterson, E. Pettersson, W. J. Peyrot, G. Pistis, D. Posthuma, S. M. Purcell, J. A. Quiroz, P. Qvist, J. P. Rice, B. P. Riley, M. Rivera, S. S. Mirza, R. Saxena, R. Schoevers, E. C. Schulte, L. Shen, J. Shi, S. I. Shyn, E. Sigurdsson, G. B. C. Sinnamon, J. H. Smit, D. J. Smith, H. Stefansson, S. Steinberg, C. A. Stockmeier, F. Streit, J. Strohmaier, K. E. Tansey, H. Teismann, A. Teumer, W. Thompson, P. A. Thomson, T. E. Thorgeirsson, C. Tian, M. Traylor, J. Treutlein, V. Trubetskoy, A. G. Uitterlinden, D. Umbricht, S. Van der Auwera, A. M. van Hemert, A. Viktorin, P. M. Visscher, Y. Wang, B. T. Webb, S. M. Weinsheimer, J. Wellmann, G. Willemsen, S. H. Witt, Y. Wu, H. S. Xi, J. Yang, F. Zhang, eQTLGen, 23andMe, V. Arolt, B. T. Baune, K. Berger, D. I. Boomsma, S. Cichon, U. Dannlowski, E. C. J. de Geus, J. R. De Paulo, E. Domenici, K. Domschke, T. Esko, H. J. Grabe, S. P. Hamilton, C. Hayward, A. C. Heath, D. A. Hinds, K. S. Kendler, S. Kloiber, G. Lewis, Q. S. Li, S. Lucae, P. F. A. Madden, P. K. Magnusson, N. G. Martin, A. M. M. Intosh, A. Metspalu, O. Mors, P. B. Mortensen, B. Müller-Myhsok, M. Nordentoft, M. M. Nöthen, M. C. O’Donovan, S. A. Paciga, N. L. Pedersen, B. W. J. H. Penninx, R. H. Perlis, D. J. Porteous, J. B. Potash, M. Preisig, M. Rietschel, C. Schaefer, T. G. Schulze, J. W. Smoller, K. Stefansson, H. Tiemeier, R. Uher, H. Völzke, M. M. Weissman, T. Werge, A. R. Winslow, C. M. Lewis, D. F. Levinson, G. Breen, A. D. Børglum, P. F. Sullivan, Major Depressive Disorder Working Group of the Psychiatric Genomics Consortium, Genome-wide association analyses identify 44 risk variants and refine the genetic architecture of major depression. Nat. Genet. 50, 668–681 (2018). 29700475 10.1038/s41588-018-0090-3 PMC5934326

