# Supplementary material for: Deep learning reveals a neurocomputational mechanism predicting depression risk in adolescents
Source: Sci Adv. 2026 Aug 7;12(32):eaed0772. doi: 10.1126/sciadv.aed0772 (PMC13450214; doi:10.1126/sciadv.aed0772)
Supplement: Supplementary file 1 [file sciadv.aed0772_sm.pdf]

Science Advances
AAAS

Supplementary Materials for

Deep learning reveals a neurocomputational mechanism predicting depression risk in adolescents

Han Lu *et al.*

Corresponding author: Qiang Luo, qluo@fudan.edu.cn

Sci. Adv. **12**, eaed0772 (2026)
DOI: 10.1126/sciadv.aed0772

This PDF file includes:

List of IMAGEN Consortium members
List of STRATIFY/ESTRA Consortium members
Supplementary Text
Figs. S1 to S15
Tables S1 to S6

IMAGEN Consortium members

Andreas Heinz (University of Tübingen; Technische Universität Dresden), Tobias Banaschewski (Heidelberg University), Gareth J. Barker (King’s College London), Arun L.W. Bokde (Trinity College Dublin), Rüdiger Brühl (Physikalisch-Technische Bundesanstalt), Sylvane Desrivières (King’s College London), Herta Flor (Heidelberg University; University of Mannheim), Hugh Garavan (University of Vermont), Penny Gowland (University of Nottingham), Antoine Grigis (Université Paris-Saclay), Herve Lemaitre (Université Paris-Saclay; Université de Bordeaux), Jean-Luc Martinot (Université Paris-Saclay; Barthélémy Durand Hospital), Marie-Laure Paillère Martinot (Université Paris-Saclay; Pitié-Salpêtrière Hospital), Eric Artiges (Université Paris-Saclay; Barthélémy Durand Hospital), Frauke Nees (Ludwig-Maximilians-Universität (LMU) in Munich), Dimitri Papadopoulos Orfanos (University of Mannheim), Luise Poustka (University Hospital Heidelberg), Michael N. Smolka (Technische Universität Dresden), Sarah Hohman (Heidelberg University), Nathalie Holz (Heidelberg University), Nilakshi Vaidya (Charité Universitätsmedizin Berlin), Henrik Walter (Charité – Universitätsmedizin Berlin), Robert Whelan (Trinity College Dublin), Gunter Schumann (Charité Universitätsmedizin Berlin; Fudan University)

STRATIFY/ESTRA Consortium members

Nilakshi Vaidya (Charité Universitätsmedizin Berlin), Zuo Zhang (King's College London; University of Birmingham), Lauren Robinson (King's College London; Oxford University), Jeanne Winterer (Charité – Universitätsmedizin Berlin; Freie Universität Berlin), Sinead King (King's College London), Yuning Zhang (University of Southampton), Gareth J Barker (King's College London), Arun L Bokde (Trinity College Dublin), Rüdiger Brühl (Physikalisch-Technische Bundesanstalt), Hedi Kebir (Charité Universitätsmedizin Berlin), Hervé Lemaître (Université Paris-Saclay), Frauke Nees (Ludwig-Maximilians-Universität (LMU) in Munich), Dimitri Papadopoulos Orfanos (University of Mannheim), Ulrike Schmidt (King's College London), Julia Sinclair (University of Southampton), Robert Whelan (Trinity College Dublin), Henrik Walter (Charité – Universitätsmedizin Berlin), Sylvane Desrivières (King's College London), Gunter Schumann (Charité Universitätsmedizin Berlin; Fudan University)

Supplementary Text

Method S1 Model Optimization and Evaluation

To determine the optimal hyperparameters for training the VAE model, we evaluated several hyperparameters including batch sizes, network structures with different hidden layers, learning rates, dropout rates, and the dimensionality of the latent layer z. Hyperparameter optimization was performed in a structured, multi-stage manner using five-fold cross-validation. For each combination, the average loss curve was obtained by averaging results across the five folds, and reconstruction accuracy for each participant was calculated using cosine similarity.

- Network structure and batch size.

We first evaluated batch sizes of 16, 32, and 64, and examined models with one hidden layer of 1,024 neurons, two hidden layers with 1,024 and 512 neurons, and three hidden layers with 1,024, 512, and 256 neurons, resulting in 9 different configurations. These hyperparameters primarily determine representational capacity and the effective use of training samples. Among these configurations, models with two hidden layers and batch sizes of 32 or 64 achieved the best performance and were therefore selected as candidates for the optimal hyperparameters (Figure S2a).

- Learning rate.

Based on these candidate architectures, we evaluated learning rates of 1e-5, 1e-4, and 1e-3, since learning rate mainly affects optimization stability and convergence behavior, and meaningful comparisons require a fixed network structure. In both candidate configurations, a learning rate of 1e-4 yielded the most stable loss curves and highest reconstruction accuracy (Figure S2b).

- Dropout rate.

We next tuned the dropout rate (0, 0.1, 0.3, 0.5) after fixing the architecture and learning rate. A dropout rate of 0.3 consistently resulted in the best performance across candidate configurations (Figure S2c).

- Latent dimensionality.

We adjusted the dimensionality of the latent variable (z = 128, 256, 512) to calibrate

the model's information bottleneck after stabilizing optimization and regularization. A latent dimensionality of 256 achieved the best performance across both candidate configurations (Figure S2d).

Among these configurations, the best performance was achieved by models using a batch size of 32 or 64, two hidden layers, a learning rate of 1e-4, a dropout rate of 0.3, and a latent dimensionality of 256; these configurations were subsequently used to compare model stability. Next, the model stability was evaluated by repeating training three times with the same hyperparameters but different random seeds. For each participant, we calculated the difference in cosine similarity to all other participants. If the model produced consistent results, the average change in cosine similarity should be zero. The model with the average change closest to zero was considered the most stable. We performed a paired t-test on the average changes between the two optimal hyperparameter candidates. The model with a batch size of 64 demonstrated greater stability compared to the one with a batch size of 32 ($p < 0.001$, Fig. S2e). Therefore, the optimal VAE hyperparameters were found to be a batch size of 64 with two hidden layers (1,024 and 512 neurons), a learning rate of 1e-4, a dropout rate of 0.3, and a latent dimensionality of 256.

To evaluate the effectiveness of the VAE model used in this study, we compared its performance with that of a traditional linear dimensionality reduction method—Principal Component Analysis (PCA). PCA was used to reduce the dimensionality of all grey matter voxels and occipital-related voxels to 256 dimensions, respectively. Paired t-tests demonstrated that the VAE yielded significantly higher reconstruction accuracy (cosine similarity) than PCA at the group level (Fig. S3). We further visualized individual-level performance for whole-brain grey matter (Fig. S4) and occipital-related voxels (Fig. S5). Representative subjects were identified by selecting the subject whose VAE reconstruction accuracy was closest to the group median across 1,332 samples. For each representative subject, we plotted the ground-truth activation maps alongside the reconstructions generated by VAE and PCA.

We also assessed whether the representations of different ROI activation patterns obtained from the trained VAE model were distinguishable. Specifically, we extracted activation patterns for different regions of interest (ROIs) based on the AAL2 atlas. Following the same procedure applied to the occipital-related network (see Methods), activations were retained only at the ROI locations while setting all other locations to zero. For each participant, this process yielded a sparse vector with 47,640 voxels, which was then input into the trained VAE model to obtain 256-dimensional latent representations. These representations were visualized using t-SNE (Fig. S6).

Method S2 Evaluation of cluster stability and group differences in a held-out sample

We conducted an additional sensitivity analysis using a train-test split to evaluate the robustness of the clustering solution and its associated behavioral effects. Participants were randomly divided into a training set (70%) and a held-out test set (30%), with stratification by data collection site to preserve site distributions across splits.

The variational autoencoder (VAE) was trained exclusively on the training set. The trained model was then used to extract 256-dimensional visual anger codes for individuals in both the training and test sets.

Within the training set, we applied k-means clustering to the visual anger codes. K-means clustering minimizes the total within-cluster sum of squared Euclidean distances between individual codes and their corresponding cluster centroids. To determine candidate numbers of clusters, we used two complementary criteria: (i) the elbow method, which evaluates the decrease in within-cluster sum of squares as the number of clusters increases, and (ii) the silhouette score, which quantifies how well each observation is matched to its own cluster relative to other clusters, with higher average silhouette values indicating better cluster separation. Both criteria suggested candidate solutions ranging from two to four clusters.

To assess clustering stability, we evaluated solutions with 2, 3, and 4 clusters using 100 repeated k-means runs with identical parameters but different random initializations. For each solution, we constructed a consensus matrix reflecting the proportion of times that each pair of participants was assigned to the same cluster across repetitions. Cluster stability was quantified using the proportion of ambiguous clustering (PAC), defined as the fraction of consensus values falling between 0.1 and 0.9; lower PAC values indicate higher clustering stability. This analysis consistently identified the two-cluster solution as the most stable configuration in the training set.

We then evaluated the generalizability of this solution in the held-out test set. For each of the 100 repeated clustering runs, test participants were assigned to clusters based on the nearest training-derived centroids. Stability analyses across the 2-4 cluster solutions again indicated that the two-cluster solution exhibited the highest stability in the test set.

Finally, using the two-cluster assignment in the test set, we examined group differences in coding efficiency and emotional symptoms. Consistent with the main analysis, individuals in the low-efficiency group showed significantly reduced coding efficiency and elevated emotional symptoms compared with those in the high-efficiency group, supporting the robustness of the primary findings.

Method S3 Information Gain Analysis and Bootstrap Resampling Procedure

For the 256-dimensional visual emotion code derived from the VAE model, we first normalized the data to the range [-1, 1]. Then, given a specified number of bins (n), we calculated the number of data points falling into each bin and converted these counts into probabilities p_i. The entropy of the visual emotion code H_{actual} was then calculated as:

$$H_{actual} = -\sum_{i=1}^{n} p_i log_2(p_i).$$

To quantify the **information gain** of the visual emotion code, we defined it as the reduction in entropy relative to a random distribution with the maximum uncertainty. Specifically, the information gain (IG) was calculated as:

$IG = H_{max} - H_{actual}$,

where $H_{max} = H_{uniform}$ represented the entropy of a uniform distribution, calculated as:

$$H_{uniform} = -\sum_{i=1}^{n} \frac{1}{n} log_2(\frac{1}{n}).$$

The comparison of information gain between two groups, referred to as the low-efficiency group and the high-efficiency group, was performed using a bootstrap resampling procedure. To mitigate the influence of individual noise, the information gain for each group was calculated based on the mean visual emotion code across participants. The bootstrap procedure consisted of the following steps:

1. Computed the overall information gain for each group:
2. Performed 1000 bootstrap iterations, where for each iteration:
   - Sampled with replacement from the subjects of the low-efficiency group and high-efficiency group to generate bootstrap samples
   - Computed the information gain for each bootstrap sample
   - Recorded the difference in information gain between the two groups:

$$\Delta IG = IG_{sample\_high} - IG_{sample\_low}$$

3. Aggregated the differences in information gain across all bootstrap iterations to form a distribution. The 95% confidence interval (CI) for the difference in information gain was determined using the 2.5th and 97.5th percentiles of the bootstrap distribution.

Finally, we assessed statistical significance by examining whether the 95% confidence interval (CI) included zero. A CI entirely above zero indicated significantly greater information gain in the high-efficiency group, while a CI entirely below zero indicated greater information gain in the low-efficiency group. If the CI included zero, no significant difference was observed.

Method S4 Visual happy code

The contrast map of happy vs. neutral faces from the IMAGEN face task at age 19 was used to measure brain activation in response to happy faces. Following the same preprocessing steps applied to angry faces (see Methods), we extracted activations for happy faces within the occipital-related network, represented as a sparse vector with 47,640 voxels per participant, with non-zero values only in the occipital-related network. These processed activations were input into the trained VAE model to generate each participant's visual happy code. As a control, we performed k-means clustering on the visual happy code. Fig. S7 shows the results of the analysis of the visual happy code.

Method S5 Construction of CRPN Contrast Maps for Angry vs. Neutral Faces

The IMAGEN face task at age 19 included four angry face videos and four neutral face videos, each lasting 18 seconds. In these videos, up to six actors (three male and three female) displayed either angry or neutral facial expressions. The videos were converted into datasets at a frame rate of 30 frames per second, resulting in 540 frames per video. Frames were used directly (i.e., no face detection/cropping was applied), because each frame contains only a single actor: within each video, each actor appears in a separate clip, and clips are concatenated sequentially. Actor identity and clip boundaries were therefore obtained via manual annotation, which also yields contiguous frame segments belonging to the same actor. Prior to entering the CRPN, each RGB frame was resized to 224 × 224 × 3 and normalized using ImageNet mean and standard deviation. Each frame was processed sequentially through the trained CRPN model to compute the probability of belonging to its corresponding emotion category (anger or neutral). Inference was performed in a frame-wise manner (each frame independently; no temporal modeling). For each actor in each video, the top 10 frames with the highest probabilities were selected. Across the four angry face videos, all six actors appeared in each video, yielding a total of 240 high-confidence angry frames. In the case of neutral face videos, one video featured only five actors, while the others had six, resulting in a total of 230 high-confidence neutral frames.

Next, the high-confidence angry frames and neutral frames were processed through the trained CRPN encoder to obtain their respective 256-dimensional representations. The CRPN encoder output was a single 256-dimensional vector per frame. The average representation of the angry frames was subtracted from that of the neutral frames to generate the CRPN angry vs. neutral faces contrast map**,** which is defined in this study as the CRPN code.

Method S6 Hierarchical mapping in CRPN

For the CRPN encoder, we used ResNet-18 as the backbone and replaced the final output layer with two fully-connected layers, each with 256 neurons. In other words, the CRPN encoder outputs a 256-dimensional representation. To investigate which layer's features from the CRPN encoder are most suitable for mapping to brain activations in the occipital-related network, we extracted the output features from the five convolutional blocks (conv1, conv2, conv3, conv4, conv5) of the ResNet-18 portion of the CRPN encoder, as well as the output features from the two newly added fully connected layers (FC1, percept). Specifically, for high-confidence angry frames, we recorded the output features of the aforementioned network layers. To ensure fair comparison across all layers, we applied PCA to reduce the feature dimensions to 200 for each layer, resulting in a 240×200 matrix for each layer. Similarly, for high-confidence neutral frames, a 230×200 matrix was obtained for each network layer. For each layer, we calculated the CRPN contrast map and compared it with the brain's visual anger code to compute the brain score. As shown in Fig. S8, the perceptual features from the complete CRPN encoder were more suitable for mapping to brain activation patterns compared to the other layers.

Method S7 Comparative Analysis of Neural Markers versus Family Stress

We compared the predictive performance of neural markers against family stress, a well-established environmental risk factor for emotional disorders. Family stress was quantified using the Development and Well-Being Assessment (DAWBA). We calculated a composite family stress score by summing the responses from 16 items within the family-stress subscale.

We used linear discriminant analysis (LDA) to predict high-risk status (symptom score > 75th percentile at age 23):

1. Family-Stress Model: Included the composite family stress score alongside basic covariates (sex, site, and handedness).
2. Neural Model: Included the identified neural markers (optimal- and over-regularized brain scores) alongside basic covariates (sex, site, and handedness), but excluded the family stress score.

The performance of both models was evaluated using a 5-fold cross-validation scheme, repeated 100 times to ensure robustness. The Area Under the Receiver Operating Characteristic Curve (AUC) was calculated for each repetition. We evaluate the significance of AUC differences between the neural model and family-stress model using paired t-tests.

Supplementary Figures

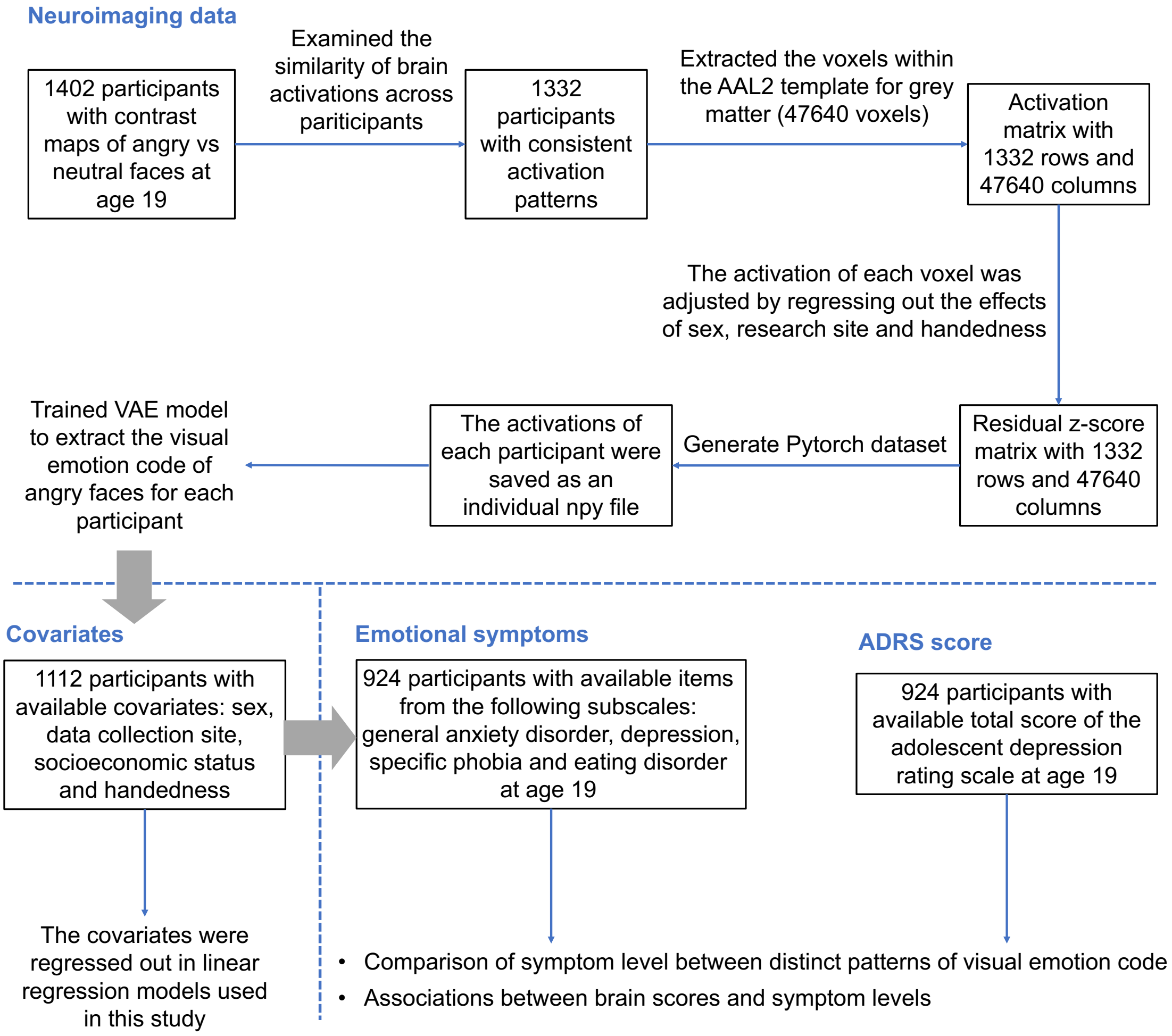

Fig. S1 Flow diagram of the participants in the IMAGEN cohort included in this study.

ADRS score means the total score of the adolescent depression rating scale.

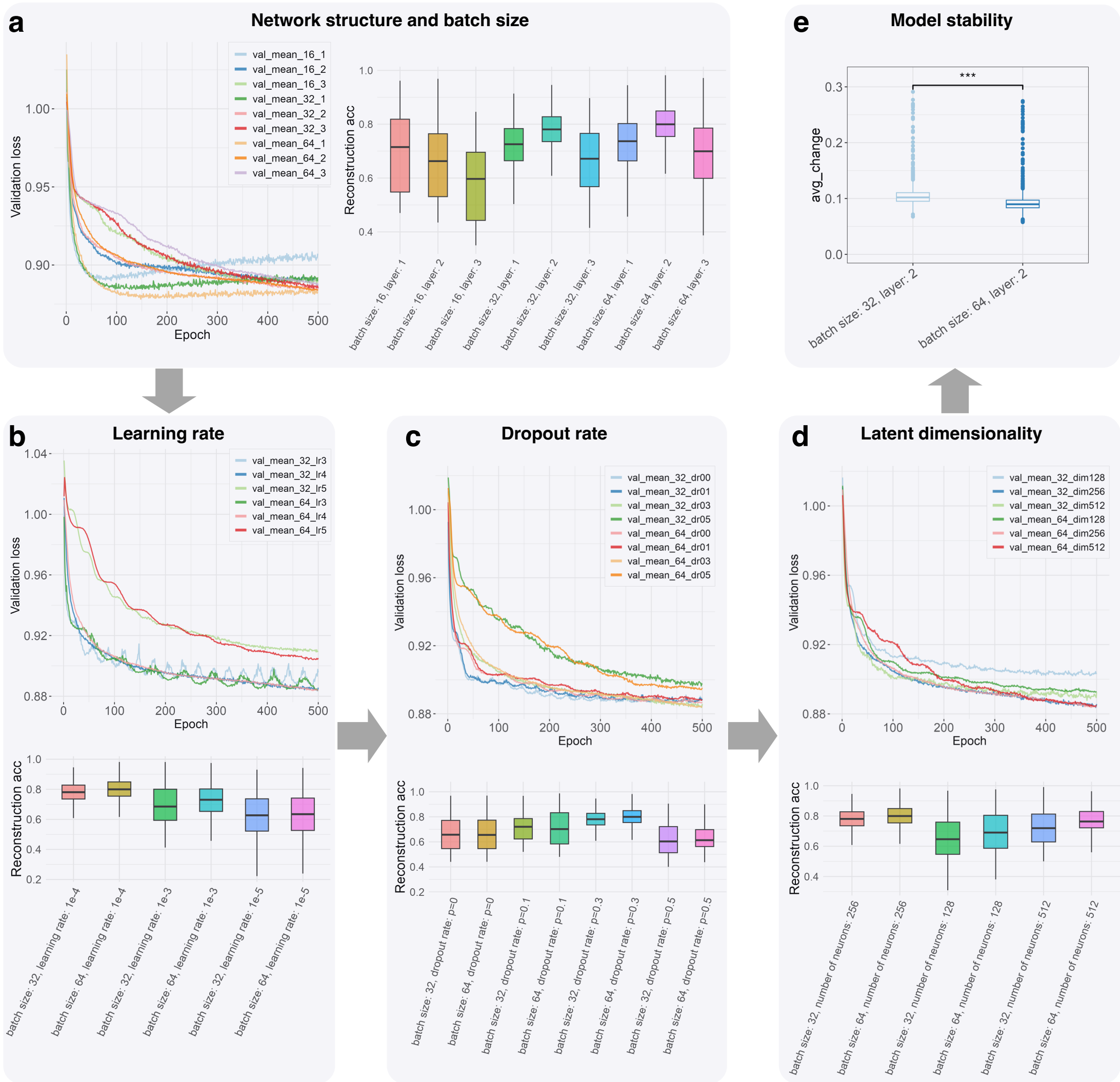

Fig. S2 VAE performance across different hyperparameters.

(**a-d**) Five-fold cross-validation was used to evaluate the VAE for different hyperparameter combinations. (**e**) The stability of the two candidate hyperparameter combinations was evaluated. *** represents p<0.001.

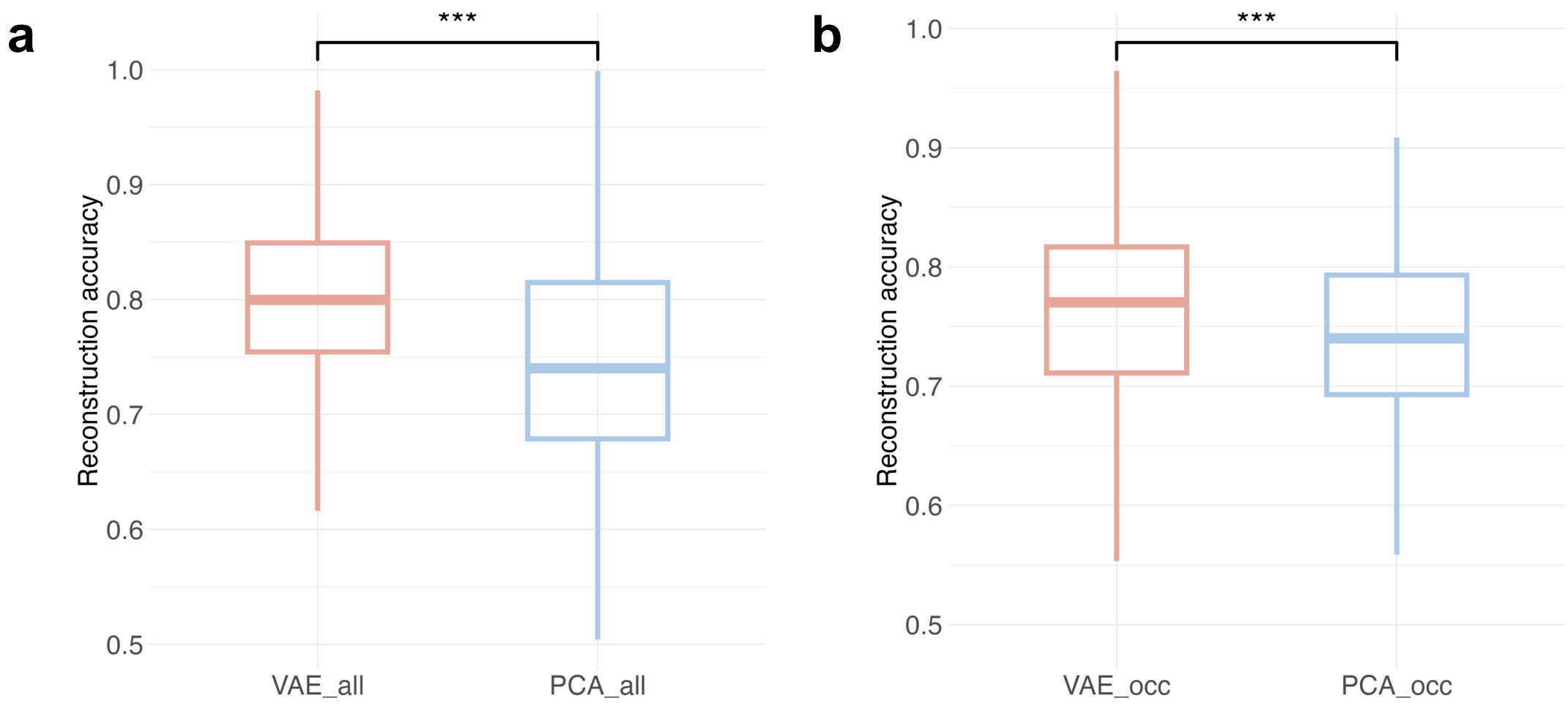

Fig. S3 Comparison of reconstruction accuracy between VAE and PCA.

(**a**) Reconstruction of all grey matter voxels (47,640 voxels). (**b**) Reconstruction of the occipital-related voxels. The VAE model consistently outperformed PCA across both voxel sets. *** represents $p<0.001$.

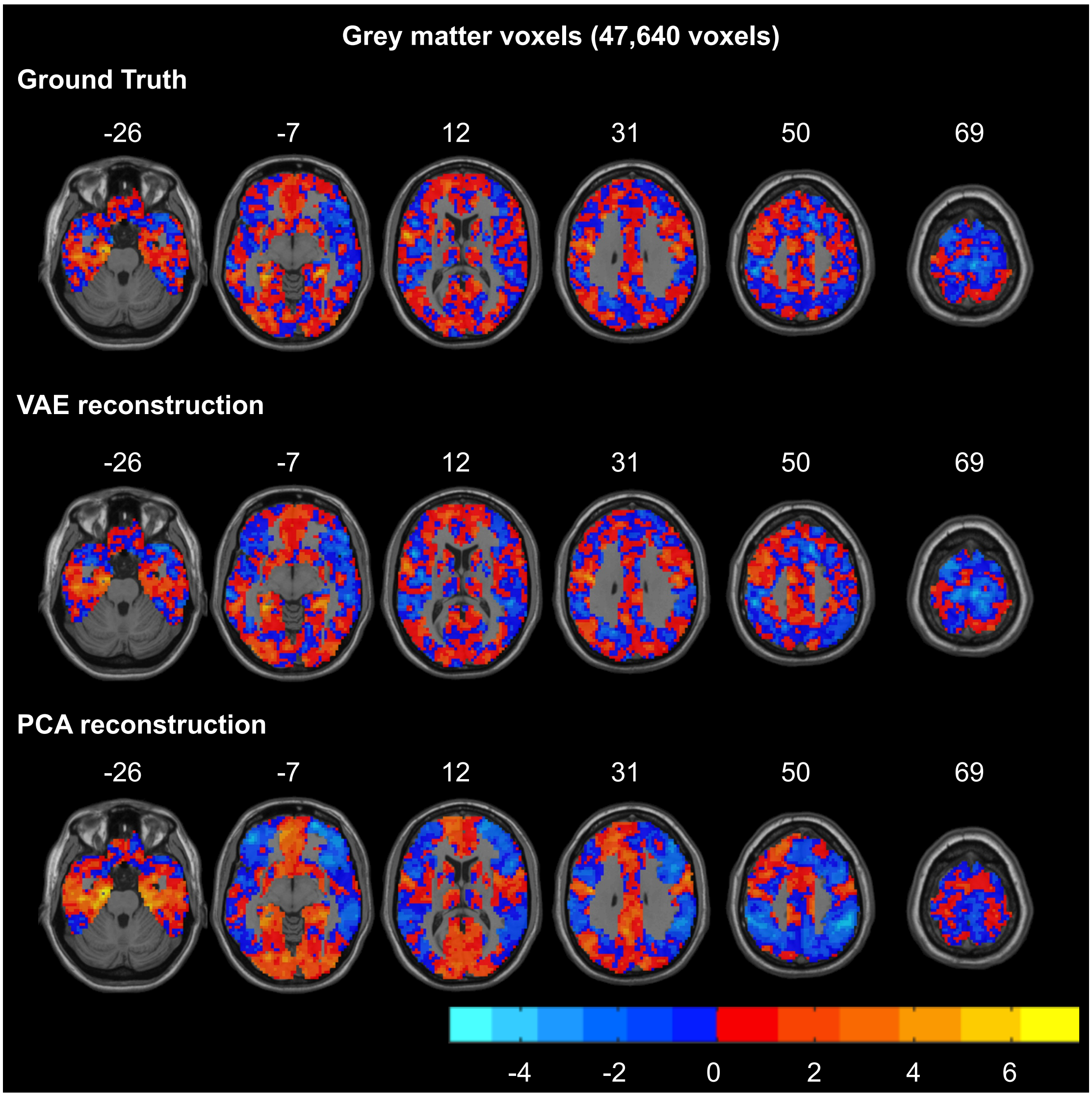

Fig. S4 Visualization of individual-level reconstruction performance for grey matter voxels.

The figure displays brain activation maps for a representative subject, selected based on the median VAE reconstruction accuracy across the entire sample (*N*=1,332). Axial slices are displayed at MNI coordinates *z*=-26, -7, 12, 31, 50, and 69.

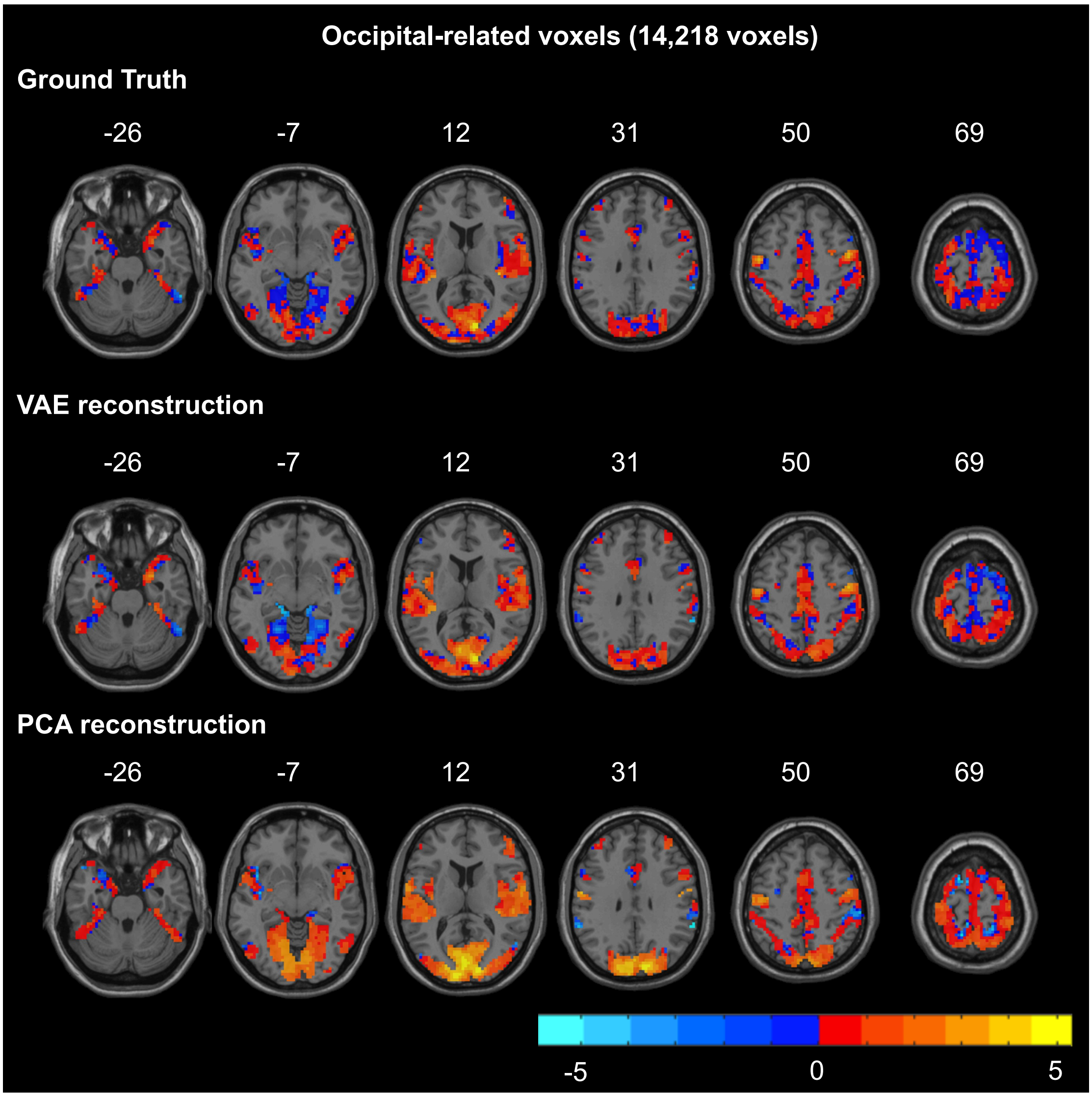

Fig. S5 Visualization of individual-level reconstruction performance for occipital-related voxels.

The figure displays brain activation maps for a representative subject, selected based on the median VAE reconstruction accuracy across the entire sample (*N*=1,332). Axial slices are displayed at MNI coordinates z=-26, -7, 12, 31, 50, and 69.

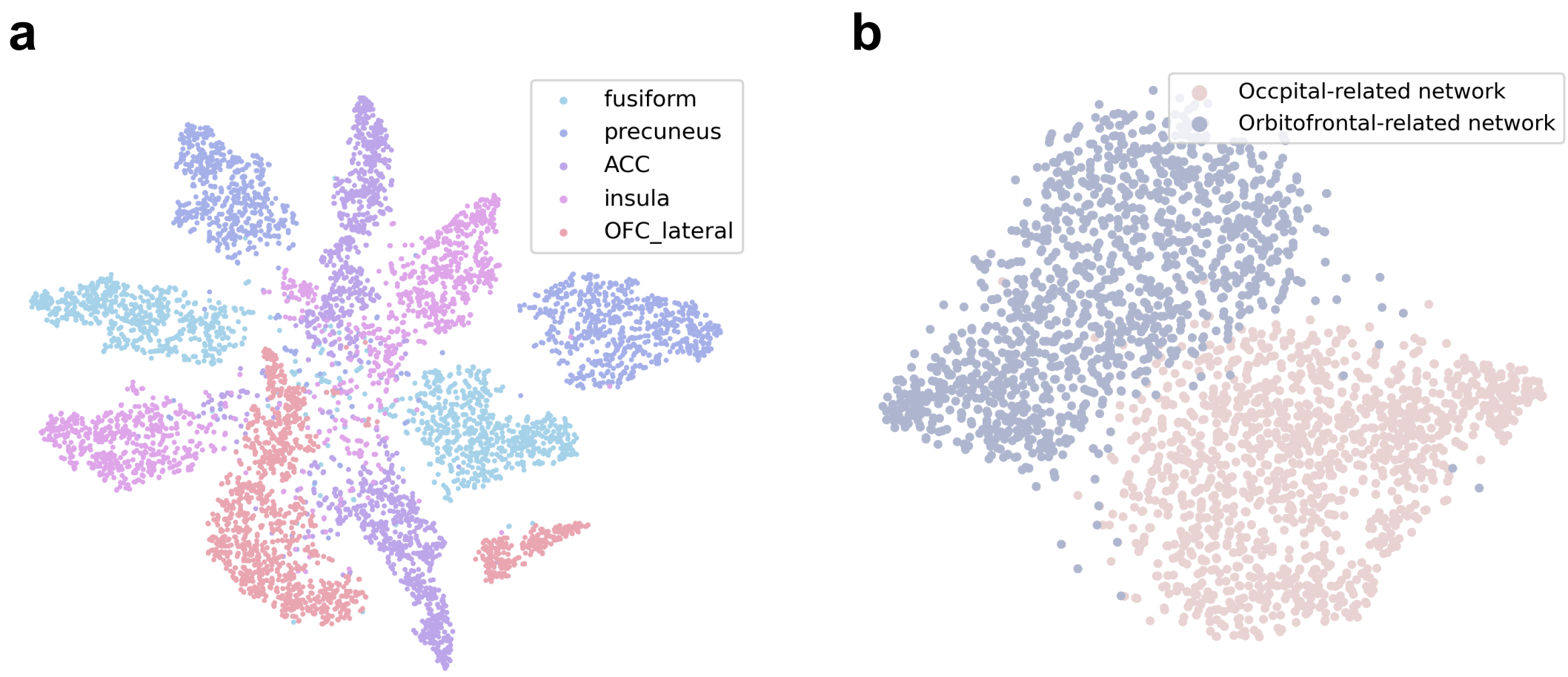

Fig. S6 The distribution of different ROI activation patterns processed through the same trained VAE.

(**a**) Different brain regions obtained based on the AAL2 template. (**b**) Two networks obtained from a previous work on the brain's facial emotion processing system (Lu et al. (16)).

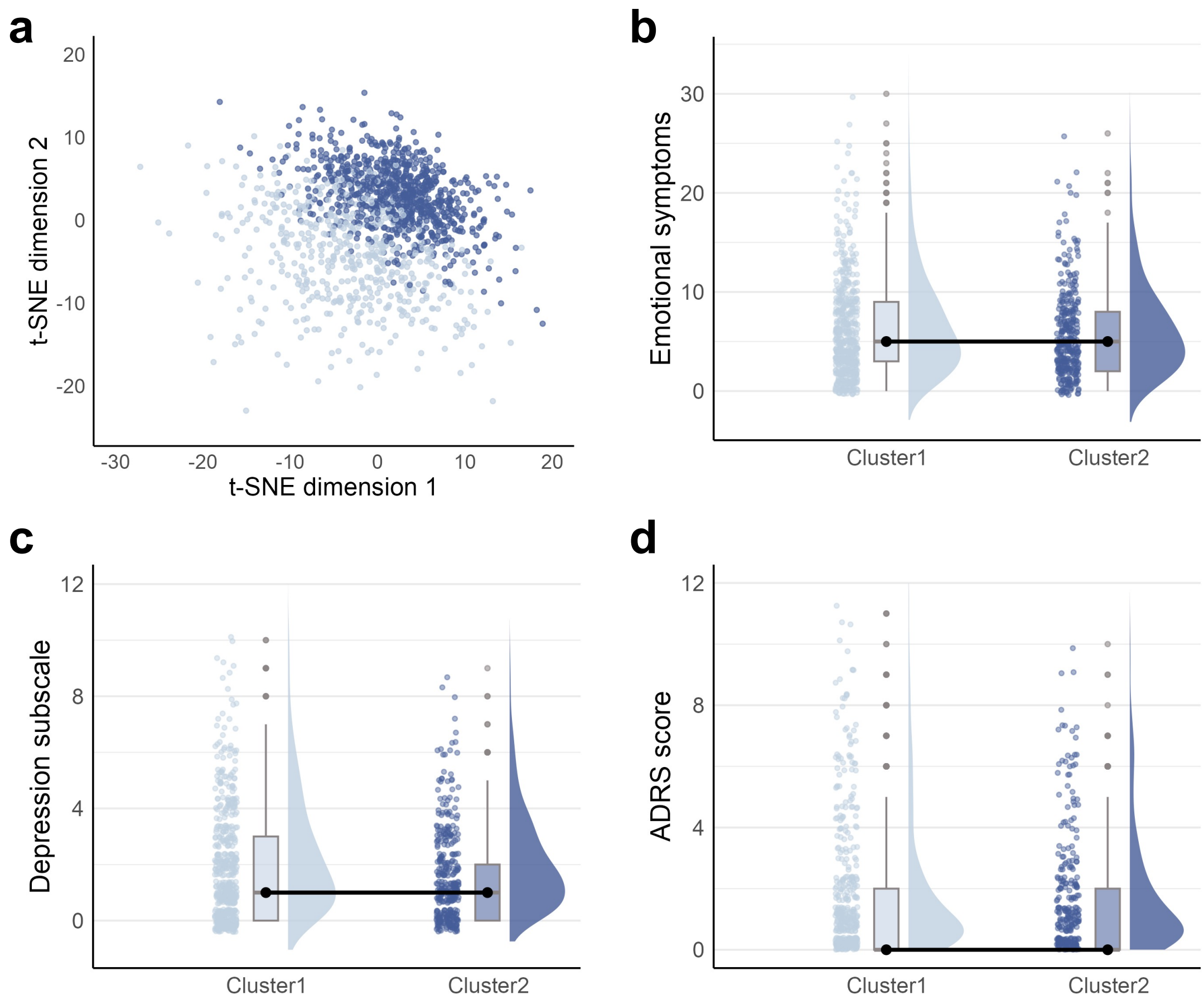

Fig. S7 Analysis of the visual happy code.

(**a**) When we set k=2 in the K-means clustering algorithm (consistent with the angry face condition), the patterns of the two clusters were indistinguishable. (**b-d**) There were no significant differences between the two clusters in terms of emotional symptoms, depression subscale, or ASRS scores.

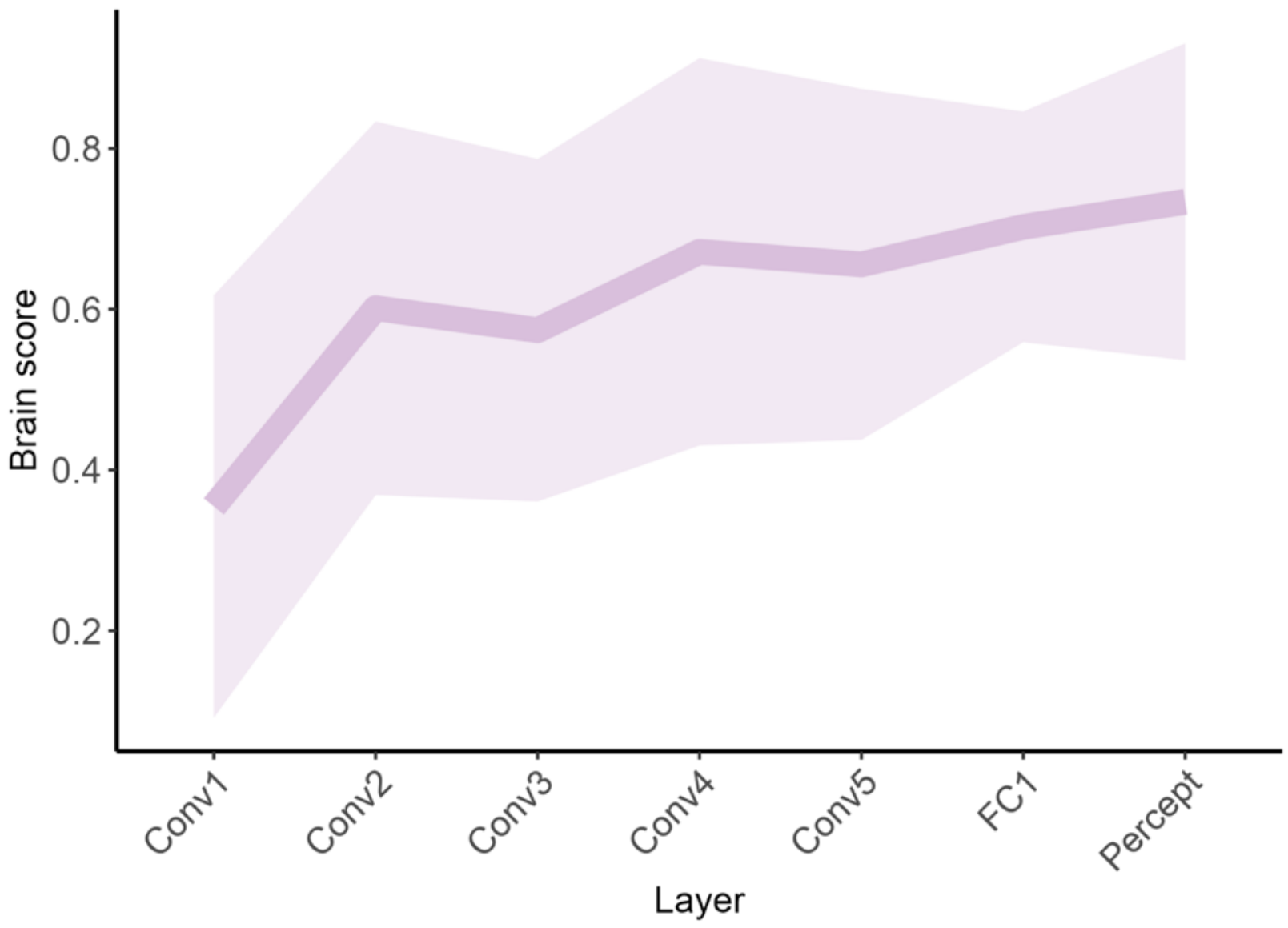

Fig. S8 Brain scores between the brain's visual anger code and different layers of the CRPN.

A higher brain score indicates a higher similarity.

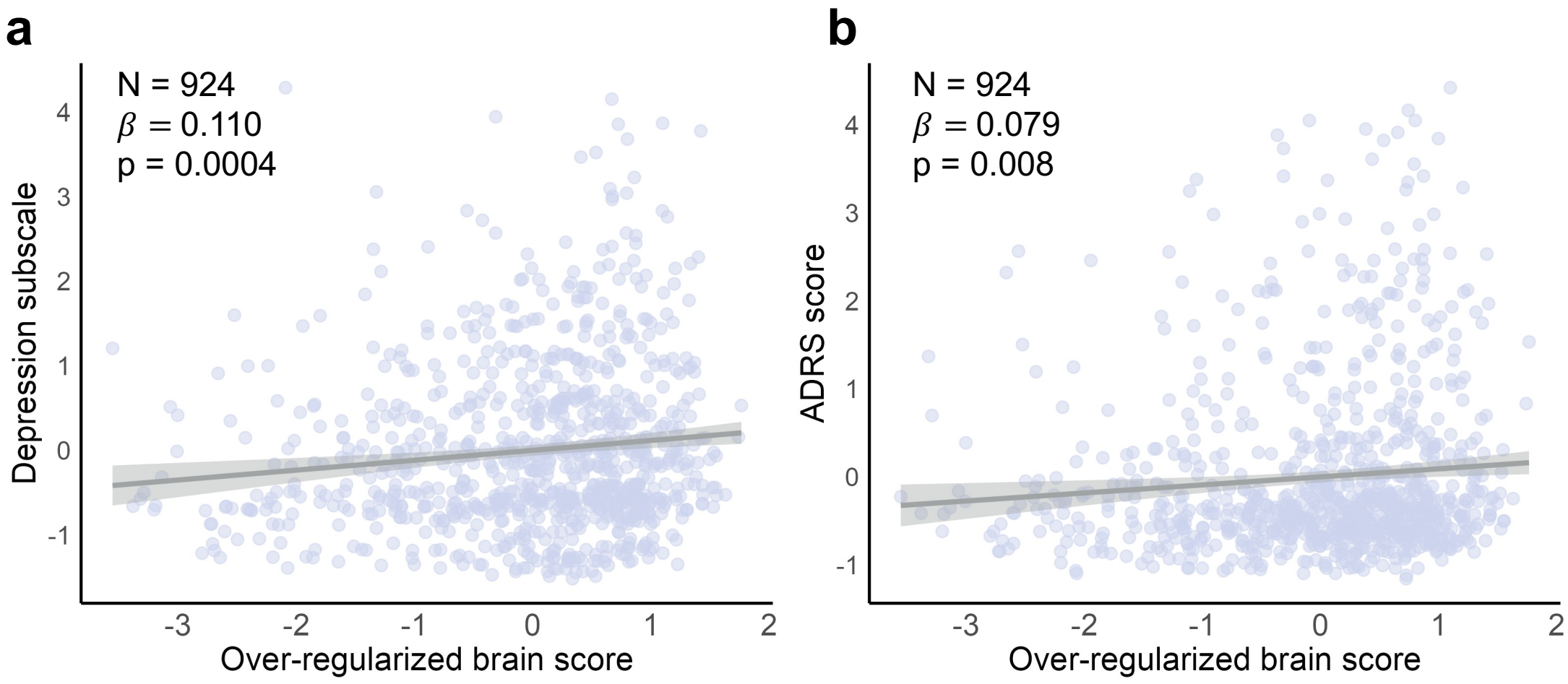

Fig. S9 The association between the over-regularized brain score and symptom levels at age 19.

(**a-b**) Over-regularized brain scores were significantly associated with both the depression subscale and ADRS scores. Plots display residual z-scores adjusted for covariates (see Methods).

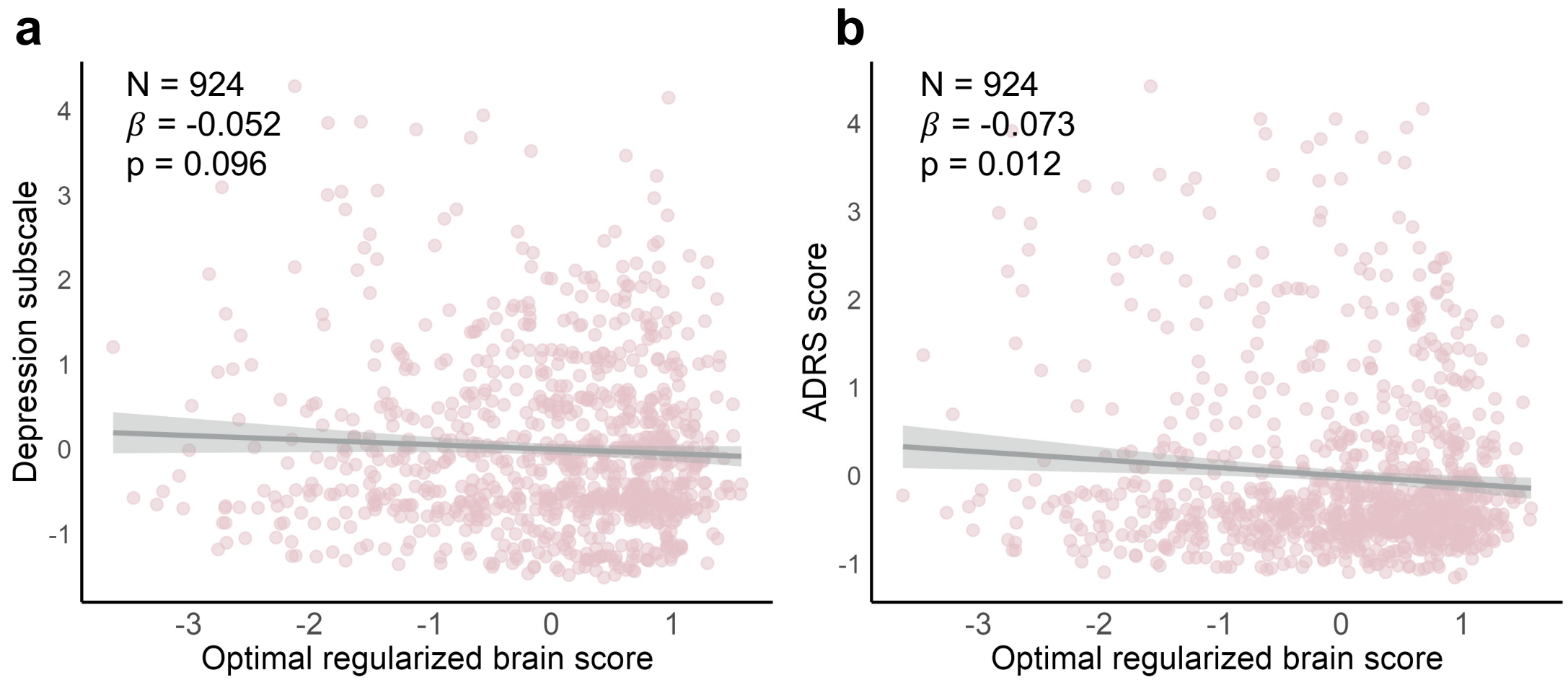

Fig. S10 Association between the optimal regularized brain score and symptom levels at age 19.

(**a**) The association between the optimal regularized brain score and the depression subscale was not statistically significant (p=0.096). (**b**) The optimal regularized brain score was significantly associated with the ADRS score (p=0.012). Plots display residual z-scores adjusted for covariates (see Methods).

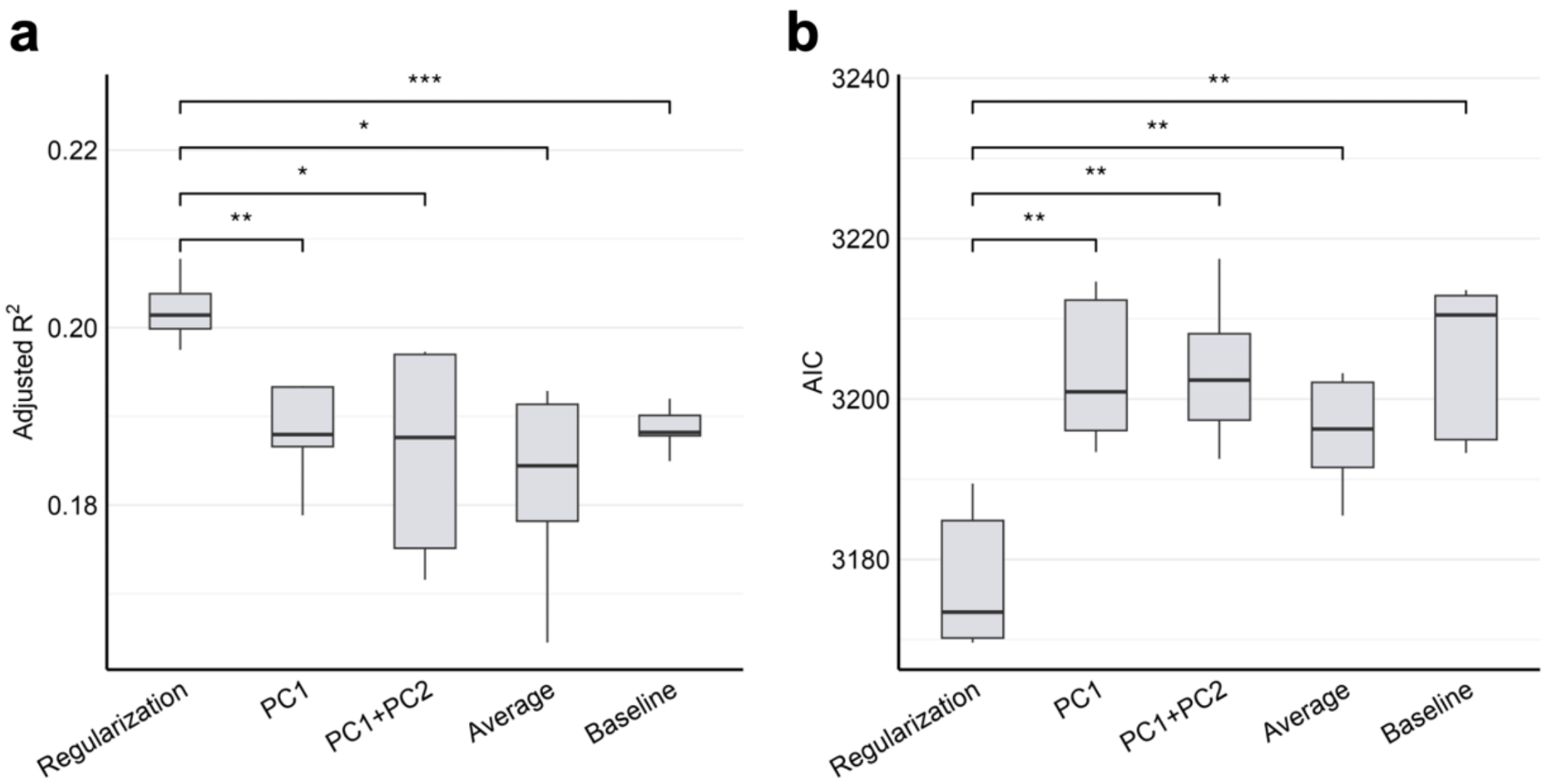

Fig. S11 Comparison of model performance across three models for predicting emotional symptoms at age 23 using data age 19.

(**a**) The regularization model exhibited a significantly higher adjusted R^2 compared to the activation models and the baseline model. (**b**) The regularization model demonstrated a significantly lower Akaike Information Criterion (AIC) relative to all other models. The baseline model used the sex, data collection site, handedness and socioeconomic status as predictors. Based on this, three variants of the activation model were developed by incorporating: (1) the first principal component (PC) of occipital-related activation, (2) the first and second PCs, and (3) the mean activation across the occipital-related regions. The regularization model extended the baseline model by additionally including both the optimal- and over-regularized scores. * p<0.05, ** p<0.01, *** p<0.001.

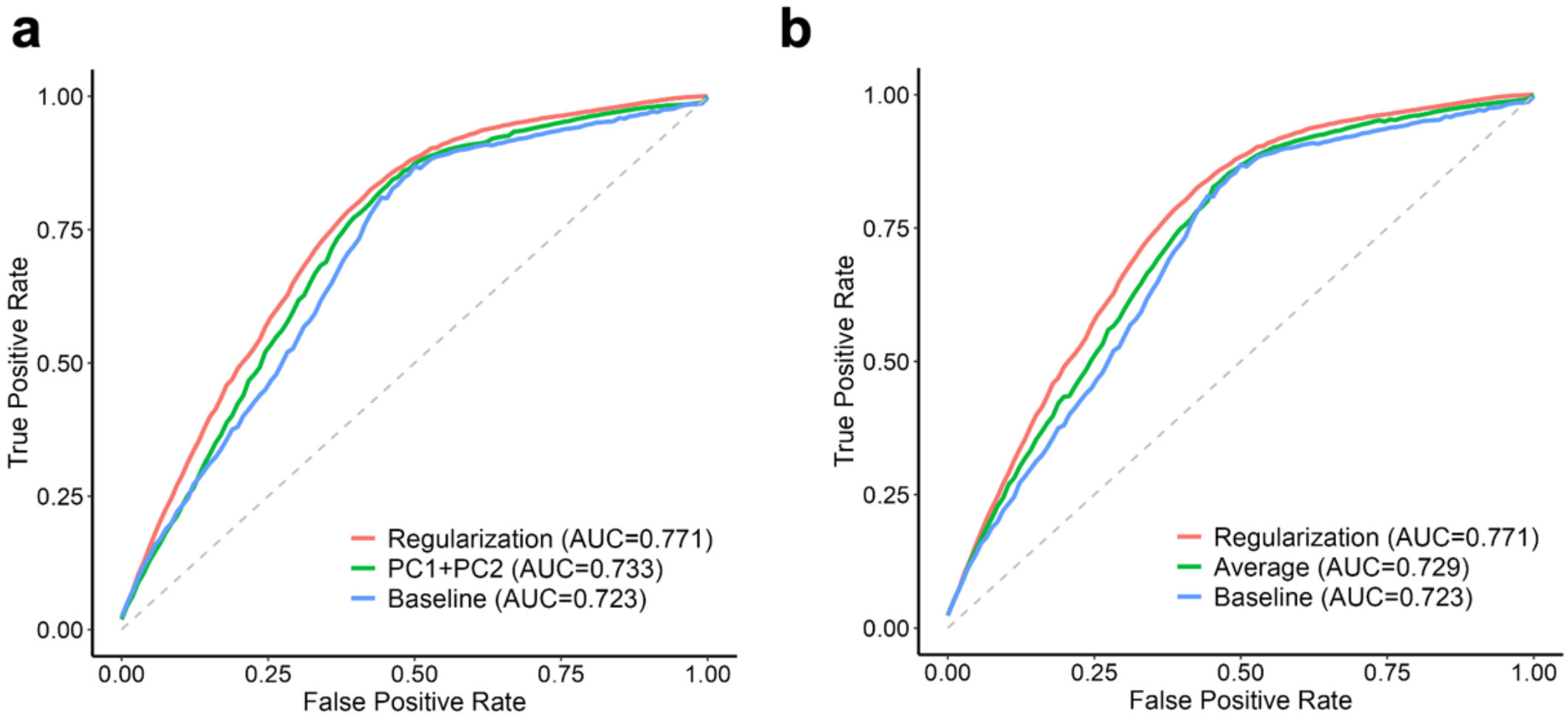

Fig. S12 Comparison of AUC across three models for predicting high-risk individuals 4 years before symptom onset.

(**a**) Performance comparison between the regularization model, the baseline model, and an alternative activation model incorporating both the first and second PCs. (**b**) Performance comparison between the regularization model, the baseline model, and an alternative activation model using the mean occipital-related activation.

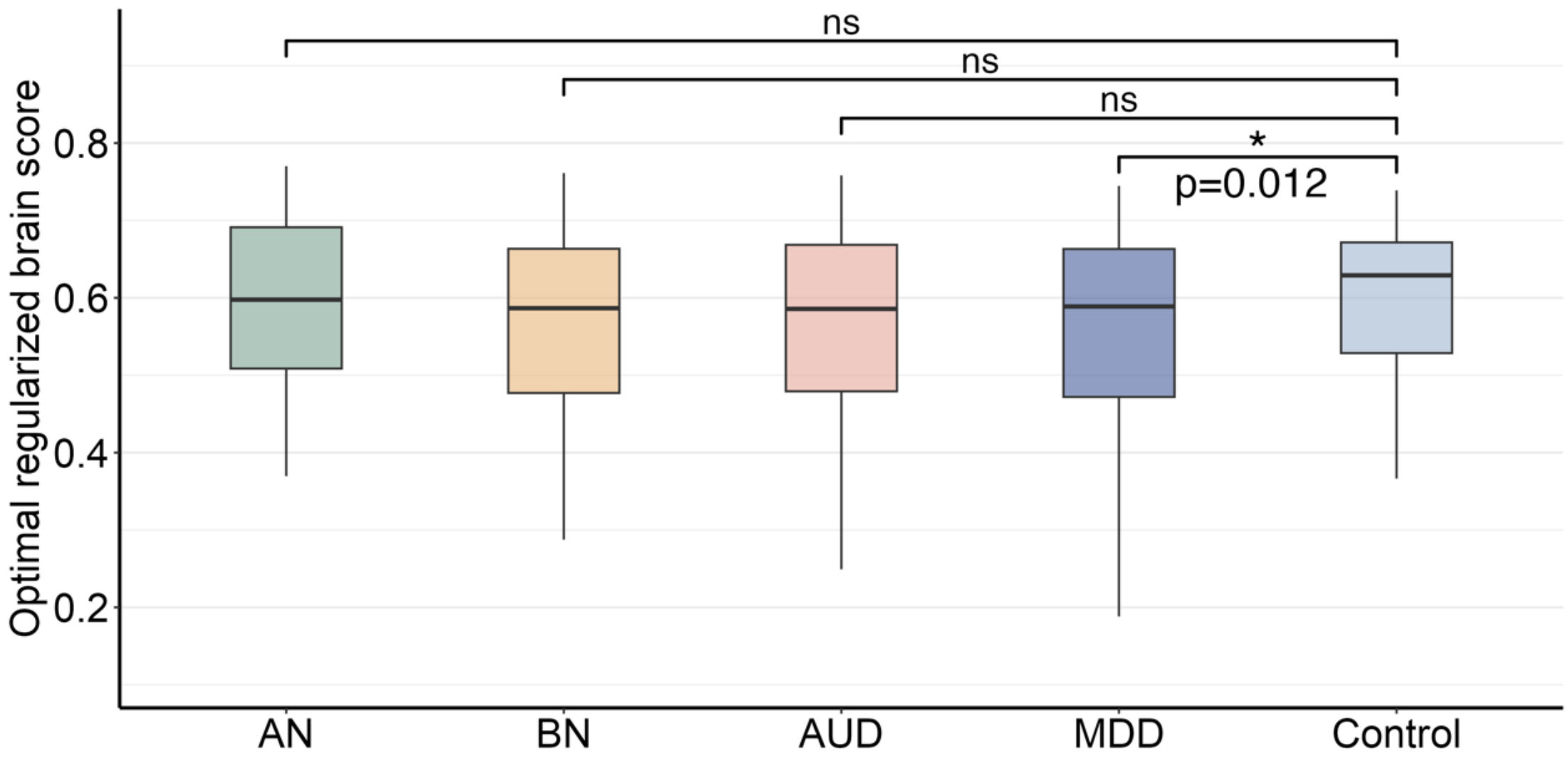

Fig. S13 Case-control comparison of the optimal-regularized brain scores.

Box plots depict the distribution of scores across the clinical groups (AN, BN, AUD, MDD) and healthy controls. The MDD group exhibited significantly lower scores compared to the control group (p=0.012). In contrast, no significant differences were observed between the control group and the AN, BN, or AUD groups (ns, not significant).

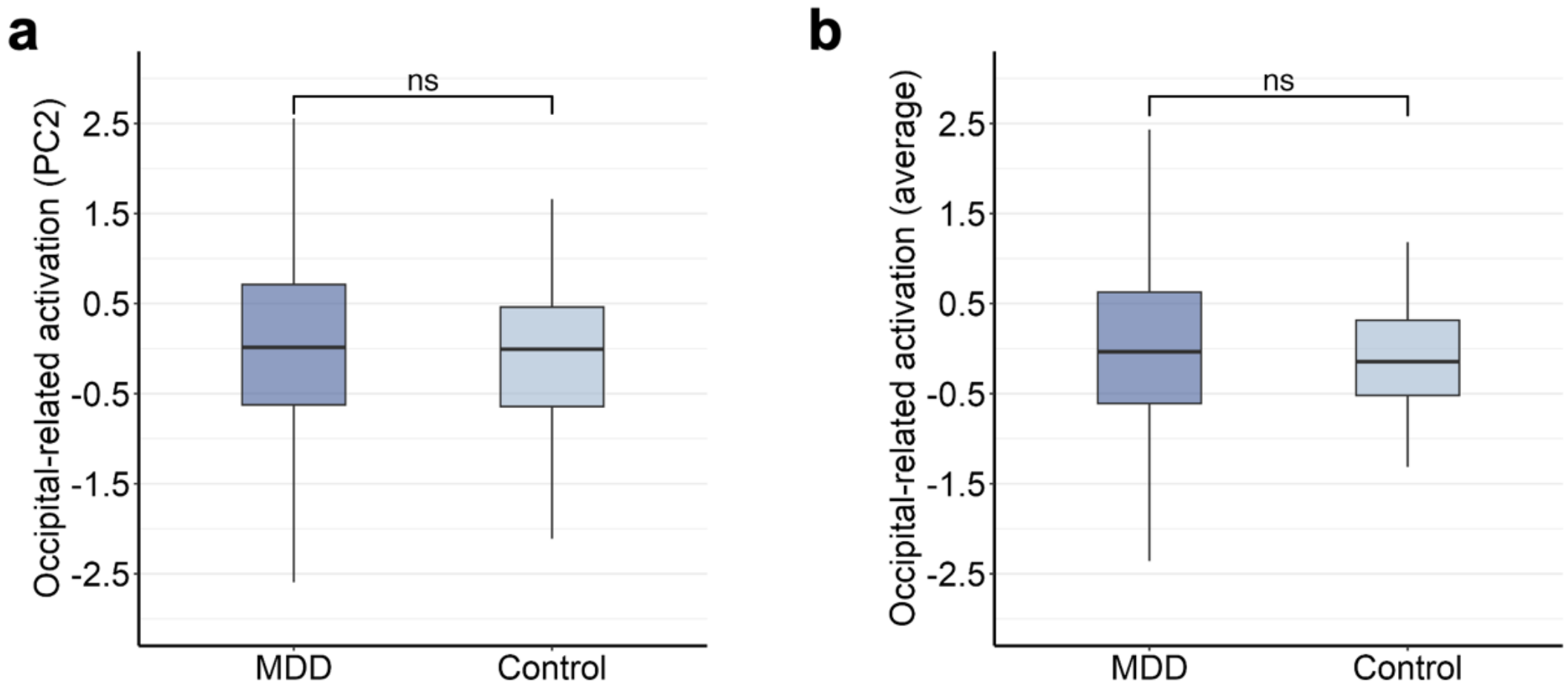

Fig. S14 Case-control comparison of the occipital-related activation metrics.

(**a**) Box plots showing the second PC of the occipital-related activation. (**b**) Box plots showing the average occipital-related activation. In both analyses, no significant differences were observed between the MDD group and the control group (ns, not significant).

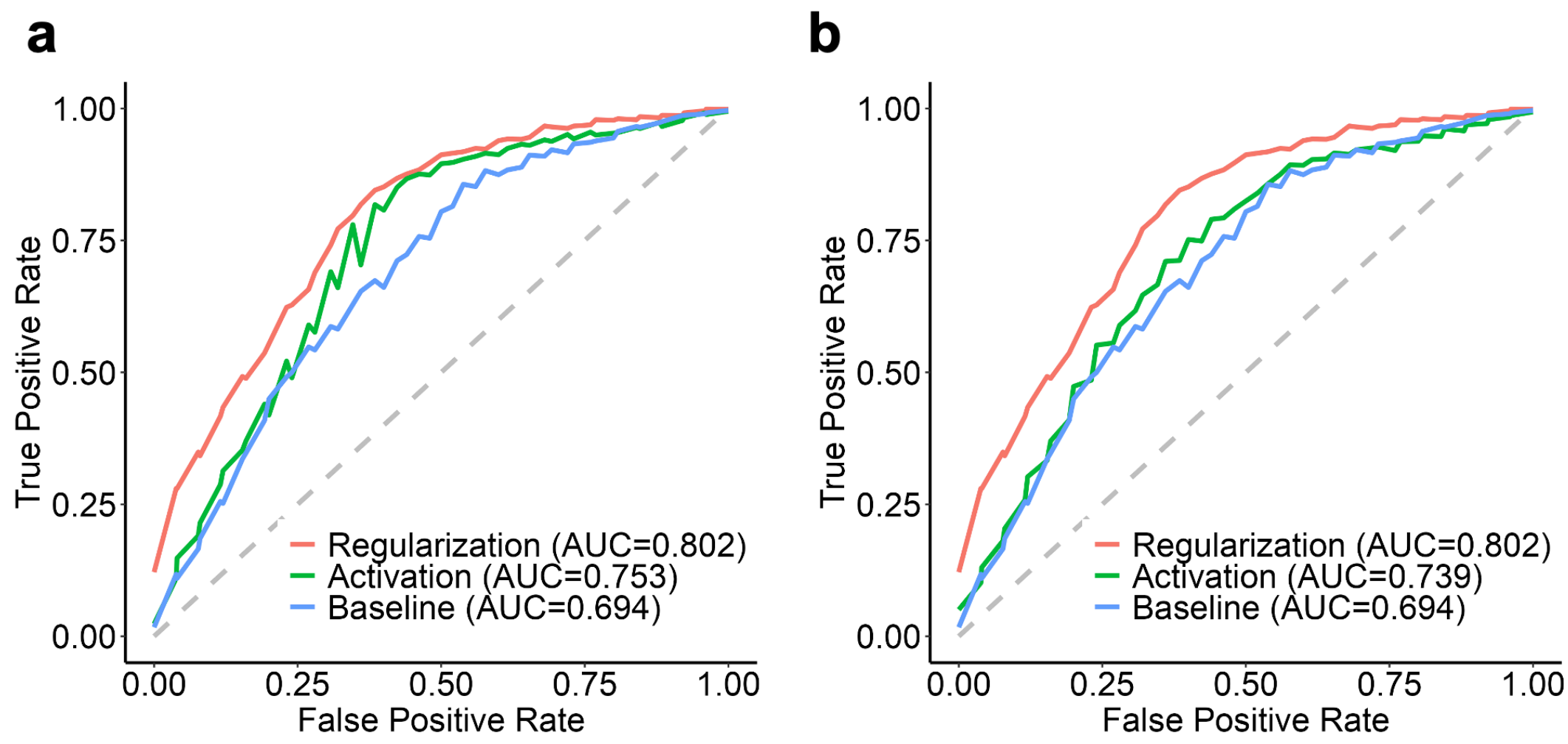

Fig. S15 Comparison of AUC across three models for classifying individuals into MDD and healthy controls.

(**a**) Performance comparison between the regularization model, the baseline model, and an alternative activation model incorporating both the first and second PCs. (**b**) Performance comparison between the regularization model, the baseline model, and an alternative activation model using the mean occipital-related activation.

Supplementary Tables

Table S1 Demographic characteristics of the samples in this study

**Variables**	**Age 19**	**Age 23**
Sex, Female, n (%)	704 (52.85%)	415 (57.24%)
Social Economic Status	2.59 (±2.40)	2.59 (±2.40)
Hand, Right, n (%)	1193 (89.56%)	1193 (89.56%)
Research Site (%)		
London	166 (12.46%)	166 (12.46%)
Nottingham	214 (16.07%)	214 (16.07%)
Dublin	122 (9.16%)	122 (9.16%)
Berlin	136 (10.21%)	136 (10.21%)
Hamburg	172 (12.91%)	172 (12.91%)
Mannheim	151 (11.34%)	151 (11.34%)
Paris	198 (14.86%)	198 (14.86%)
Dresden	173 (12.99%)	173 (12.99%)
Emotional symptoms	5.95 (±4.78)	5.22 (±4.35)
ADRS score	1.31(±2.12)	NA

Numbers of subjects are presented as integers (percentage), and quantitative measurements are presented as mean values ± standard deviations.

Table S2 Robustness of the performance advantage across data collection sites.

Data collection site	ΔS	95%CI
London	0.108	[0.055, 0.115]
Nottingham	0.098	[0.011, 0.118]
Dublin	0.104	[0.0004, 0.116]
Berlin	0.087	[0.011, 0.116]
Hamburg	0.106	[0.026, 0.117]
Mannheim	0.114	[0.043, 0.118]
Paris	0.099	[0.009, 0.115]
Dresden	0.105	[0.001, 0.118]

The brain score differences (ΔS) and 95%CI indicate that the CRPN model consistently outperformed the PerceptNet model across all data collection sites.

Table S3 Group differences in brain scores related to CRPN code under varying regularization intensities

Regularization intensity	ΔBrain score	95%CI	Significance
0	0.049	[-0.001, 0.107]	ns
0.1	0.034	[-0.023, 0.097]	ns
0.2	0.015	[-0.01, 0.058]	ns
0.3	-0.073	[-0.115, -0.034]	Low-efficiency
0.4	-0.206	[-0.245, -0.157]	Low-efficiency
0.5	-0.132	[-0.172, -0.068]	Low-efficiency
0.6	-0.047	[-0.092, 0.014]	ns
0.7	-0.032	[-0.081, 0.037]	ns
0.8	0.063	[0.023, 0.124]	High-efficiency
0.9	0.126	[0.091, 0.162]	High-efficiency
1	0.161	[0.121, 0.202]	High-efficiency
1.1	0.101	[0.048, 0.149]	High-efficiency
1.2	0.077	[0.029, 0.132]	High-efficiency
1.3	0.032	[0.0003, 0.084]	High-efficiency
1.4	0.021	[-0.02, 0.076]	ns
1.5	-0.011	[-0.059, 0.056]	ns
1.6	-0.056	[-0.109, 0.015]	ns
1.7	-0.114	[-0.16, -0.046]	Low-efficiency
1.8	-0.125	[-0.164, -0.067]	Low-efficiency
1.9	-0.162	[-0.219, -0.131]	Low-efficiency
2	-0.168	[-0.219, -0.122]	Low-efficiency

ΔBrain score: difference between high- and low-efficiency groups.

Table S4 Sex-stratified associations between brain scores and emotional symptoms

	Girl			Boy		
Regularized brain score	β	95%CI	p	β	95%CI	p
Over-	0.128	[0.048, 0.208]	0.002	0.083	[-0.011, 0.178]	0.085
Optimal-	-0.084	[-0.163, -0.004]	0.040	-0.064	[-0.159, 0.031]	0.186

The results show significant associations in females and directionally consistent but smaller-magnitude effects in males.

Table S5 The items of emotional symptoms used in this study

General anxiety disorder (7 items)	Depression (8 items)
Panic.attacks.in.last.4.weeks (DAWBA_Self)	Dep.sad (DAWBA_Self)
Gena.ever.worries (DAWBA_Self)	Dep.irritable (DAWBA_Self)
Gena.specific.or.generalised (DAWBA_Self)	Dep.loss.of.interest (DAWBA_Self)
Gena.excessive.worry (DAWBA_Self)	Dep.recent.talk.of.dsh (DAWBA_Self)
Many worries (SDQ_Self)	Dep.dsh.recently (DAWBA_Self)
Many fears (SDQ_Self)	Dep.dsh.ever (DAWBA_Self)
Anxious in new situations (SDQ_Self)	Headache/stomach ache (SDQ_Self)
	Unhappy (SDQ_Self)
**Specific Phobia (13 items)**	**Eating disorder (5 items)**
Specific fear of: animals (DAWBA_Self)	Eat: feels fat when isnt (DAWBA_Self)
Specific fear of: storms, thunder, heights (DAWBA_Self)	Eat: ashamed of eating habits (DAWBA_Self)
Specific fear of: dark (DAWBA_Self)	Eat: deliberate vomiting (DAWBA_Self)
Specific fear of: loud noises (DAWBA_Self)	Eat: eating concerns intefere a lot (DAWBA_Self)
Specific fear of: blood, infection, injury (DAWBA_Self)	Eat: blames self a lot for overeating (DAWBA_Self)
Specific fear of: dentists, doctors (DAWBA_Self)	
Specific fear of: vomiting, choking, diseases (DAWBA_Self)	
Specific fear of: types of transport (DAWBA_Self)	
Specific fear of: enclosed spaces (DAWBA_Self)	
Specific fear of: toilets (DAWBA_Self)	
Specific fear of: people who look unusual (DAWBA_Self)	
Specific fear of: monsters etc (DAWBA_Self)	
Specific fear of: other things (DAWBA_Self)	

Items were selected from the self-reported Development and Well-Being Assessment (DAWBA_Self) and the Strengths and Difficulties Questionnaire (SDQ_Self).

Table S6 Demographic and Psychological Differences Between Included and Excluded Participants

Variables	Included samples	Excluded samples	statistics	p value
Sex, Male, n(%)	628 (47.15%)	29 (41.43%)	χ_1^2 =0.87	0.35
Hand, Right, n(%)	1193 (89.56%)	63 (90%)	χ_1^2 =0.01	0.91
Social Economic Status	2.59 (±2.39)	2.46 (±2.53)	t=0.35	0.73
Data collection site (%)			χ_7^2 =4.46	0.73
London	166 (12.46%)	10 (14.29%)		
Nottingham	214 (16.07%)	13 (18.57%)		
Dublin	122 (9.16%)	9 (12.86%)		
Berlin	136 (10.21%)	4 (5.71%)		
Hamburg	172 (12.91%)	9 (12.86%)		
Mannheim	151 (11.34%)	10 (14.29%)		
Paris	198 (14.86%)	7 (10%)		
Dresden	173 (12.99%)	8 (11.43%)		
Head motion	0.13 (±0.08)	0.26 (±0.31)	t=-3.47	0.0009
Emotional symptoms (Age 19)	5.86 (±4.99)	6.04 (±4.56)	t=-0.61	0.54

Numbers of subjects are presented as integers (percentage), and quantitative measurements are presented as mean values ± standard deviations.
